# Efficacy of opioids for traumatic pain in the emergency department: a systematic review and Bayesian network meta-analysis

**DOI:** 10.3389/fphar.2023.1209131

**Published:** 2023-07-27

**Authors:** Yawen Fu, Qiang Liu, Hu Nie

**Affiliations:** ^1^ Department of Emergency, West China Hospital of Sichuan University, Chengdu, Sichuan, China; ^2^ West China Xiamen Hospital of Sichuan University, Xiamen, Fujian, China

**Keywords:** opioid, traumatic pain, emergency department, network meta-analysis, efficacy

## Abstract

**Aim:** To systematically assess and rank the efficacy of opioid medications for traumatic pain in the emergency department in terms of pain relief, adverse events and rescue analgesia.

**Methods:** Four databases were systematically searched until 26 September 2022: PubMed, Embase, Cochrane Library, and Web of Science. Outcomes were pain relief, adverse events (dizziness, hypotension, pruritus, sedation), and rescue analgesia. For each outcome, network plots were drawn to exhibit direct and indirect comparisons, and rank probabilities were utilized to rank the efficacy of different opioids.

**Results:** Twenty studies of 3,040 patients were eligible for this network meta-analysis. According to the rank probabilities, the top three analgesic medications for pain relief may be sufentanil (78.29% probability of ranking first), buprenorphine (48.54% probability of ranking second) and fentanyl (53.25% probability of ranking third); buprenorphine (31.20%), fentanyl (20.14%) and sufentanil (21.55%) were least likely to cause dizziness; the top three analgesic medications which were least likely to cause hypotension were buprenorphine (81.64%), morphine (45.02%) and sufentanil (17.27%); butorphanol (40.56%), morphine (41.11%) and fentanyl (14.63%) were least likely to cause pruritus; the top three medications which were least likely to cause sedation were hydrocodone + acetaminophen (97.92%), morphine (61.85%) and butorphanol (55.24%); patients who received oxycodone (83.64%), butorphanol (38.31%) and fentanyl (25.91%) were least likely to need rescue analgesia in sequence.

**Conclusion:** Sufentanil, buprenorphine and fentanyl may be superior to other opioid medications in terms of pain relief and the incidence of dizziness, hypotension and pruritus, which might be selected as opioid analgesics for traumatic pain in the emergency setting.

## Introduction

Pain is a common complaint among trauma patients in the emergency department (Dijkstra et al., 2014). Up to 70% of pre-hospital patients and 91% of emergency patients report traumatic pain (Dißmann et al., 2018). The satisfaction with pain management is low among trauma patients (Ahmadi et al., 2016). The lack of good pain treatment for trauma patients in emergency settings further leads to physical and psychological stress, which affects the treatment and rehabilitation of trauma, and incurs poor quality of life (Kariman et al., 2011; Porter et al., 2018).

Analgesic therapy is an effective strategy for pain management in the emergency department (Abu-Snieneh et al., 2022), among which opioids are the most commonly used drugs for pain (Kim et al., 2016). Opioids are usually required to relieve acute moderate to severe pain (Abdolrazaghnejad et al., 2018), and plenty of opioids are available, such as morphine, hydrocodone, buprenorphine, oxycodone and fentanyl (Trescot et al., 2008). A previous randomized controlled trial (RCT) reported that for patients with severe traumatic pain in the emergency department, intranasal sufentanil was not inferior to intravenous IV) morphine in alleviating pain in the first 30 min (Blancher et al., 2019). Vahedi et al. (Vahedi et al., 2019) found that fentanyl reduced pain faster than morphine, and fewer patients needed rescue analgesia after fentanyl in the emergency setting. Another trial proposed that despite effectiveness, intranasal fentanyl may not be a suitable analgesic choice since it resulted in hypotension and dizziness in patients using fentanyl + tramadol *versus* those using tramadol only (Chew and Shaharudin, 2017). At present, for patients with traumatic pain in the emergency department, it is still unclear which of these opioid analgesics (in addition to the above opioids) is more effective. There was a network meta-analysis conducted to evaluate the efficacy of different kinds of analgesics (non-opioid analgesics, combination therapies, nonsteroidal anti-inflammatory drugs (NSAIDs), and opioids) for patients with traumatic musculoskeletal pain in the emergency department (Yin et al., 2021). Thus, in order to help clinical decision-making on opioid analgesics, a comprehensive study is required to provide evidence related to traumatic pain management in the emergency setting.

The objective of this network meta-analysis was to systematically assess and rank the efficacy of opioid medications for traumatic pain in the emergency department in terms of pain relief, adverse events and rescue analgesia, based on both direct and indirect evidence.

## Methods

### Search strategy

Four databases were systematically searched by two authors (YW Fu and Q Liu) independently: PubMed, Embase, Cochrane Library, and Web of Science. The last search was conducted on 26 September 2022. English search terms were as follows: “Opioid” OR “Opioids” OR “Opiate” OR “Opiates” OR “Opium” OR “Papaveretum” OR “Omnopon” OR “Pantopon” OR “Morphine” OR “Morphia” OR “MS Contin” OR “Oramorph SR” OR “Duramorph” OR “Hydromorphone” OR “Dihydromorphinone” OR “Hydromorphon” OR “Dilaudid” OR “Hydrocodone “OR “Hydrocodon” OR “Dihydrocodeinone” OR “Dicodid” OR “Hycodan” OR “Oxymorphone” OR “Numorphan” OR “Oxycodone” OR “Dihydrone” OR “Eucodal” OR “Oxycontin” OR “Fentanyl” OR “Fentanil” OR “Phentanyl” OR “Fentanest” OR “Sublimaze” OR “Fentora” OR “Sufenta” OR “Heroin” OR “Meperidine” OR “Pethidine” OR “Dolosal” OR “Dolcontral” OR “Dolantin” OR “Demerol” OR “Lidol” OR “Lydol” OR “Operidine EPJ I” OR “Levorphanol” OR “Levorphan” OR “Codeine” OR “Tramadol” OR “Tramundin” OR “Zydol” OR “Adolonta” OR “Contramal” OR “Methadone” OR “Dolophine” OR “Metadol” OR “Symoron” OR “Methadose” OR “Phenadone” OR “Physeptone” OR “Amidone” OR “Methaddict” OR “Buprenorphine” OR “Buprenex” OR “Subutex” OR “Pentazocine” OR “Talwin” OR “Fortral” OR “Butorphanol” OR “Moradol” OR “Stadol” OR “Torbugesic” OR “Nalbuphine” OR “Nubain” OR “Dezocine” AND “acute trauma*” OR “trauma pain*” OR “traumatic pain*” OR “traumatic injur*” OR “acute pain*” OR “acute injur*” OR “acute wound*“. The references of relevant meta-analyses were manually retrieved to avoid missing qualified studies. Titles and abstracts of the retrieved studies were screened primarily, and full texts were used for subsequent screening. This network meta-analysis was performed following the Preferred Reporting Items for Systematic Reviews and Meta-Analysis (PRISMA) extension statement for network meta-analyses (Hutton et al., 2015).

### Study selection

Inclusion criteria included: 1) studies on patients with traumatic pain in the emergency department (population); 2) studies on opioids such as morphine, fentanyl, tramadol, oxycodone, sufentanil, and codeine (intervention and comparator); 3) studies on the following outcomes: pain relief, adverse events (dizziness, hypotension, pruritus, sedation), and rescue analgesia (outcome); 4) RCTs, cohort studies, and non-randomized controlled trials (study design). Pain relief was defined as the difference in pain scores between before treatment and after treatment, indicating the degrees of pain reduction.

Exclusion criteria included: 1) studies on a mixed population, such as a population of trauma patients and patients with other acute pain; 2) studies with a control group using non-opioid drugs alone, such as acetaminophen, ketamine, lidocaine, and ibuprofen; 3) studies on animal experiments; 4) studies with incomplete or un-extractable data; 5) case reports, meeting abstracts, letters, reviews, meta-analyses; 6) non-English publications.

### Data extraction and quality assessment

Two independent authors (YW Fu and Q Liu) extracted the following data from the included studies: first author, year of publication, country, study design, study population (indication), drug administration and type, dose, number of participants, sex (male/female), age (years), pain score, trauma type, follow-up time (FU), and outcome. Disagreements were resolved through discussion with another author (H Nie).

For the quality evaluation of RCTs, the Cochrane Collaboration’s tool for risk of bias assessment was used (Higgins et al., 2011). Six domains (selection bias, performance bias, detection bias, attrition bias, reporting bias, and other bias) were evaluated by this tool, and the risk of bias was classified as low, unclear or high. The Methodological Index for Non-Randomized Studies (MINORS) was applied to measure the quality of non-randomized studies (Slim et al., 2003), which had 12 evaluation items in total, each with a score of 0–2 (0: not reported; 1: reported but inadequate; 2: reported and adequate). A MINORS score of 1–12 was regarded as low quality and 13–24 as high quality. The quality of cohort studies was assessed with the modified Newcastle-Ottawa scale (NOS). This scale had a total score of 9, with 0–3 as poor quality, 4–6 as fair quality, and 7–9 as good quality (Stang, 2010). The evidence quality of the network meta-analysis was estimated using the Grading of Recommendations Assessment, Development, and Evaluation (GRADE) approach (Guyatt et al., 2011), which evaluated the overall quality of evidence across the following domains for each outcome: risk of bias, inconsistency, indirectness, imprecision, and other considerations. The quality of evidence was graded as high, moderate, low or very low.

### Statistical analysis

The network meta-analysis allows assessment of multiple interventions in a single analysis via combining direct and indirect evidence. Direct evidence means evidence from studies directly comparing interventions A and B (head-to-head comparison) and is an estimate of relative effects between A and B. Indirect evidence refers to the evidence obtained from paths with one or more common comparators. For instance, when there are no studies that directly compare A and B (direct evidence), an indirect comparison between A and B can be made if both are compared with C in studies (indirect evidence) (Rouse et al., 2017; Mahil et al., 2020; Papakonstantinou et al., 2020; Devall et al., 2021). This network meta-analysis was carried out using a Bayesian framework and a Monte Carlo Markov Chain (MCMC) model. The number of model chains was 4, the number of initial iterations was 20,000, the number of updated iterations was 50,000, and the step size was 1. Heterogeneity referred to the overall degree of difference in the same pair of comparisons. The I^2^ statistic was the main indicator of statistical heterogeneity, with values < 25%, 25%–50% and >50% signifying low, moderate and high heterogeneity, respectively. Consistency referred to the statistical consistency between direct and indirect effect sizes for the same comparison. The deviation information criterions (DICs) of the consistency model and the non-consistency model were compared, and a small value suggested a better fit. The absolute value of the difference in the DICs within 5 denoted consistency between indirect and direct evidence.

Network plots were drawn to exhibit direct and indirect comparisons for each outcome. A larger node indicated a larger sample size for the medication represented by the node, while a thicker line indicates a larger number of studies for comparison of the medications at both ends of the line. Forest plots and league tables were depicted to illustrate the effects of opioids on the outcomes. For pain relief, weighted mean differences (WMDs) and 95% credibility intervals (CrIs) were reported; for adverse events and rescue analgesia, relative risks (RRs) and 95%CrIs were displayed. Rank probabilities were utilized to rank the efficacy of different opioids for each outcome. STATA 15.1 (Stata Corporation, College Station, TX, United States), RevMan 5.4 (The Nordic Cochrane Centre, The Cochrane Collaboration, Copenhagen, Denmark) and R 4.1.3 (R Foundation for Statistical Computing, Vienna, Austria) software was adopted for statistical analysis.

## Results

### Study characteristics

**FIGURE 1:**
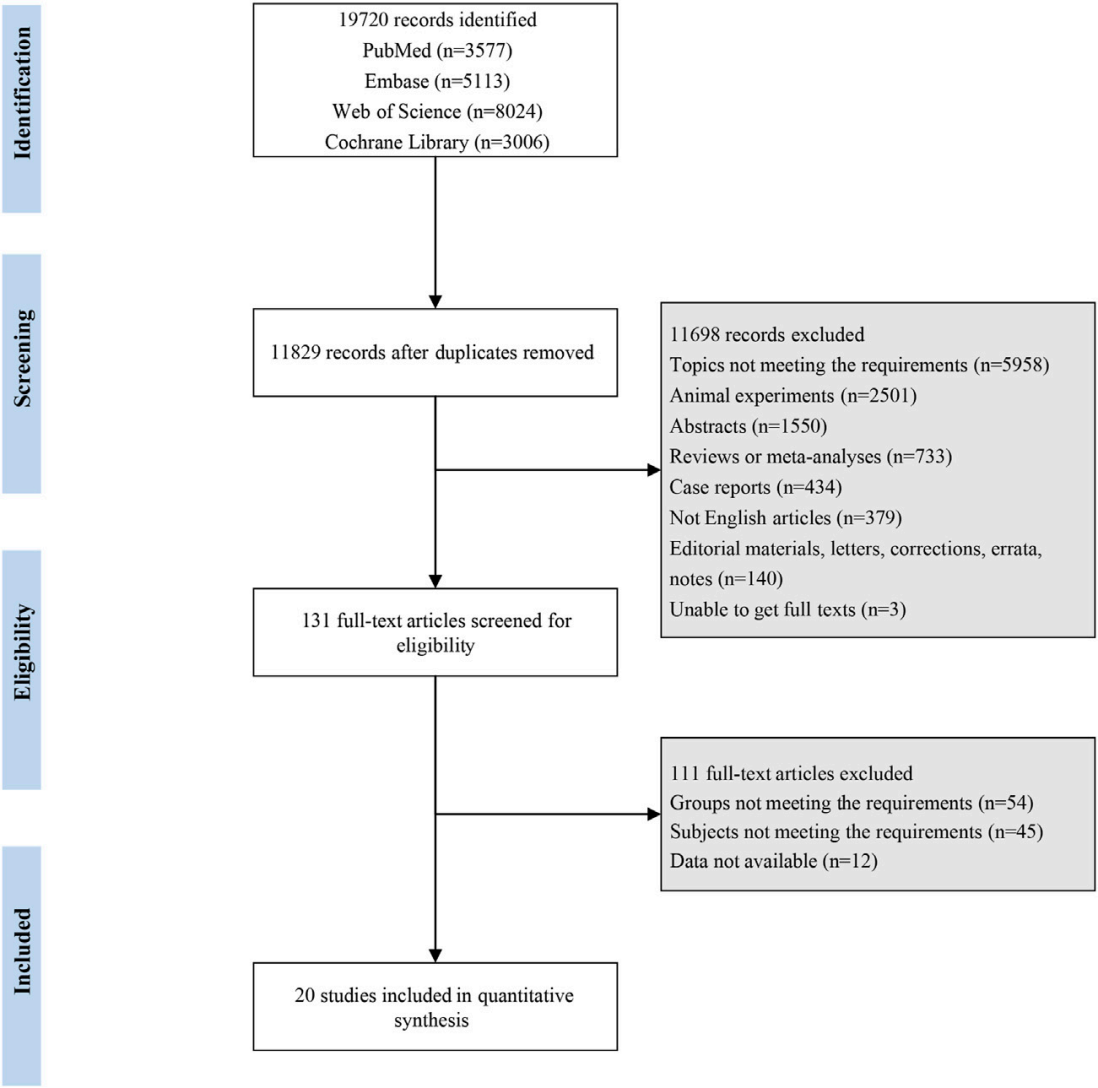
Flowchart of study selection.

A total of 19,720 were retrieved from PubMed (n = 3,577), Embase (n = 5,113), Cochrane Library (n = 8,024), and Web of Science (n = 3,006). After deleting duplicates (n = 7,891), and excluding ineligible studies based on titles and abstracts (n = 11,698) and on full texts (n = 111), 20 studies (Turturro et al., 1998; Vergnion et al., 2001; Miller and Ernst, 2004; Soysal et al., 2004; Marco et al., 2005; Hewitt et al., 2007; Bounes et al., 2010; Shear et al., 2010; Jalili et al., 2012; Smith et al., 2012; Wenderoth et al., 2013; Shervin et al., 2014; Zare et al., 2014; Chang et al., 2017; Chew and Shaharudin, 2017; Eizadi et al., 2018; Pan et al., 2018; Blancher et al., 2019; Vahedi et al., 2019; Bijur et al., 2021) of 3,040 patients were eventually included in this network meta-analysis. Figure 1 shows the flow chart of study screening. These included studies were published from 1998 to 2021, covering 7 countries. The basic characteristics of the included studies are illustrated in Supplementary Table S1. Fourteen medications were involved. There were 17 RCTs, 1 cohort study, and 2 non-randomized controlled trials.

### Quality assessment

**FIGURE 2:**
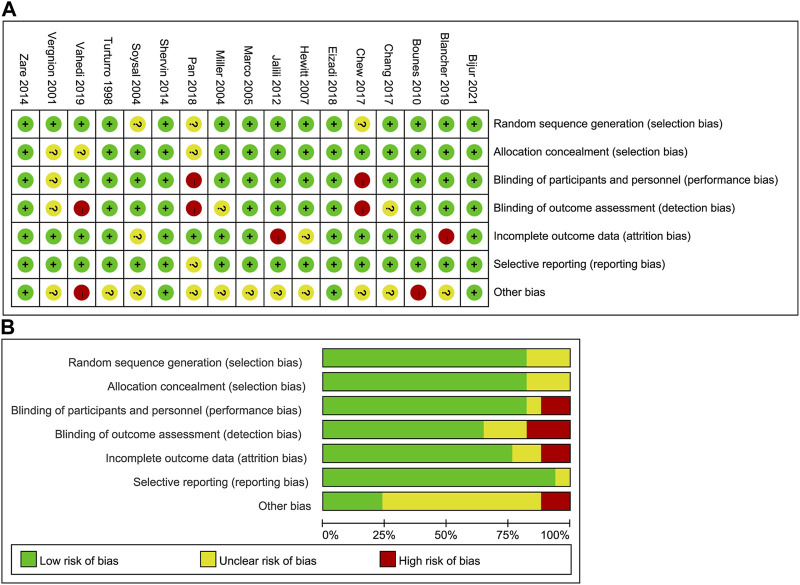
Assessment of risk of bias in the included RCTs. **(A)** Risk of bias summary; **(B)** risk of bias graph. RCT, randomized controlled trial.

For 17 RCTs, the overall risk of bias was low. Figure 2 exhibits the assessment of risk of bias. One cohort was of medium quality, and two non-randomized controlled trials were of high quality. The quality of evidence was moderate for pain relief and dizziness, and was low for hypotension, pruritus, sedation, and rescue analgesia. Quality of evidence evaluation for this network meta-analysis is shown by Supplementary Table S2 in detail.

### Network meta-analysis for pain relief

The effect of opioid medications on pain relief was investigated in 13 studies of 2,225 patients, involving 11 medications: buprenorphine, odeine + acetaminophen, fentanyl, fentanyl + tramadol, hydrocodone + acetaminophen, morphine, oxycodone, oxycodone + acetaminophen, sufentanil, tramadol, and tramadol + acetaminophen. Morphine and hydrocodone + acetaminophen were used in more patients than other medications. There were more studies for direct comparisons between morphine and fentanyl, and between oxycodone + acetaminophen and hydrocodone + acetaminophen (Supplementary Figure S1).

According to the forest plot, sufentanil relieved significantly more pain than morphine (pooled WMD = 0.70, 95%CrI: 0.01, 1.40) (Supplementary Figure S2). The league table showed that in contrast to buprenorphine (pooled WMD = -2.86, 95%CrI: −4.51, −1.21) or fentanyl (pooled WMD = −2.69, 95%CrI: −4.22, −1.15), fentanyl + tramadol was significantly less efficacious in pain relief. Hydrocodone + acetaminophen had a significantly reduced effect on pain relief than buprenorphine (pooled WMD = −0.98, 95%CrI: −1.91, −0.05) or fentanyl (pooled WMD = -0.81, 95%CrI: −1.52, −0.09). Oxycodone was inferior to buprenorphine (pooled WMD = −1.17, 95%CrI: −2.32, −0.03) or fentanyl (pooled WMD = −1.00, 95%CrI: −1.98, −0.02) in alleviating pain. Sufentanil exhibited a significantly greater impact on pain relief than codeine + acetaminophen (pooled WMD = 1.23, 95%CrI: 0.23, 2.23), fentanyl + tramadol (pooled WMD = 3.27, 95%CrI: 1.59, 4.95), hydrocodone + acetaminophen (pooled WMD = 1.38, 95%CrI: 0.40, 2.37), morphine (pooled WMD = 0.70, 95%CrI: 0.015, 1.40), and oxycodone (pooled WMD = 1.57, 95%CrI: 0.39, 2.76). Tramadol was significantly less effective in pain relief than buprenorphine (pooled WMD = −3.88, 95%CrI: −5.32, −2.43), fentanyl (pooled WMD = −3.71, 95%CrI: −5.02, −2.39), or sufentanil (pooled WMD = −4.28, 95%CrI: −5.76, −2.81). Tramadol + acetaminophen reduced significantly less pain than sufentanil (pooled WMD = −1.16, 95%CrI: −2.25, −0.06) (Table 1). Based on the rank probability, the top three analgesic medications for pain relief may be sufentanil (78.29%), buprenorphine (48.54%) and fentanyl (53.25%) in sequence (Table 2).

**TABLE 1:** League table of various opioid drugs for different outcomes in trauma patients from the emergency department.

Pain relief											
	Buprenorphine	Codeine + acetaminophen	Fentanyl	Fentanyl + tramadol	Hydrocodone + acetaminophen	Morphine	Oxycodone	Oxycodone + acetaminophen	Sufentanil	Tramadol	Tramadol + acetaminophen
Buprenorphine	Buprenorphine	−0.83 (−1.78, 0.12)	−0.17 (−0.81, 0.46)	−2.86 (−4.51, −1.21)	−0.98 (−1.91, −0.05)	−0.30 (−0.91, 0.32)	−1.17 (−2.32, −0.03)	−0.43 (−1.19, 0.34)	0.40 (−0.52, 1.32)	−3.88 (−5.32, −2.43)	−0.76 (−1.81, 0.29)
Codeine + acetaminophen	0.83 (−0.12, 1.78)	Codeine + acetaminophen	0.66 (−0.08, 1.4)	−2.03 (−3.5, −0.57)	−0.15 (−0.70, 0.40)	0.53 (−0.19, 1.25)	−0.34 (−1.55, 0.87)	0.40 (−0.16, 0.97)	1.23 (0.23, 2.23)	−3.05 (−4.28, −1.82)	0.07 (−0.66, 0.8)
Fentanyl	0.17 (−0.46, 0.81)	−0.66 (−1.4, 0.08)	Fentanyl	−2.69 (−4.22, −1.15)	−0.81 (−1.52, −0.09)	−0.12 (−0.28, 0.03)	−1.00 (−1.98, −0.02)	−0.25 (−0.73, 0.23)	0.58 (−0.13, 1.28)	−3.71 (−5.02, −2.39)	−0.59 (−1.45, 0.28)
Fentanyl + tramadol	2.86 (1.21, 4.51)	2.03 (0.57, 3.5)	2.69 (1.15, 4.22)	Fentanyl + tramadol	1.89 (0.52, 3.24)	2.57 (1.03, 4.09)	1.69 (−0.11, 3.5)	2.44 (0.97, 3.89)	3.27 (1.59, 4.95)	−1.02 (−1.81, −0.22)	2.1 (0.66, 3.55)
Hydrocodone + acetaminophen	0.98 (0.05, 1.91)	0.15 (−0.4, 0.7)	0.81 (0.09, 1.52)	−1.89 (−3.24, −0.52)	Hydrocodone + acetaminophen	0.68 (−0.02, 1.38)	−0.19 (−1.39, 1.00)	0.56 (0.02, 1.09)	1.38 (0.40, 2.37)	−2.9 (−4.0, −1.8)	0.22 (−0.27, 0.71)
Morphine	0.3 (−0.31, 0.91)	−0.53 (−1.25, 0.19)	0.12 (−0.03, 0.28)	−2.57 (−4.09, −1.03)	−0.68 (−1.38, 0.02)	Morphine	−0.87 (−1.84, 0.10)	−0.13 (−0.58, 0.32)	0.70 (0.015, 1.40)	−3.58 (−4.89, −2.28)	−0.46 (−1.31, 0.39)
Oxycodone	1.17 (0.03, 2.32)	0.34 (−0.87, 1.55)	1 (0.02, 1.98)	−1.69 (−3.5, 0.11)	0.19 (−1, 1.39)	0.87 (−0.09, 1.84)	Oxycodone	0.74 (−0.32, 1.81)	1.57 (0.39, 2.76)	−2.71 (−4.33, −1.09)	0.41 (−0.87, 1.7)
Oxycodone + acetaminophen	0.43 (−0.34, 1.19)	−0.4 (−0.97, 0.16)	0.25 (−0.23, 0.73)	−2.44 (−3.89, −0.97)	−0.55 (−1.09, −0.01)	0.13 (−0.32, 0.58)	−0.74 (−1.81, 0.32)	Oxycodone + acetaminophen	0.83 (0, 1.65)	−3.46 (−4.68, −2.23)	−0.33 (−1.05, 0.39)
Sufentanil	−0.4 (−1.32, 0.52)	−1.23 (−2.23, −0.23)	−0.58 (−1.28, 0.13)	−3.27 (−4.95, −1.59)	−1.38 (−2.37, −0.4)	−0.7 (−1.39, −0.01)	−1.57 (−2.76, −0.39)	−0.83 (−1.65, 0)	Sufentanil	−4.28 (−5.76, −2.81)	−1.16 (−2.25, −0.06)
Tramadol	3.88 (2.43, 5.32)	3.05 (1.82, 4.28)	3.71 (2.39, 5.02)	1.02 (0.23, 1.81)	2.9 (1.8, 4)	3.58 (2.28, 4.89)	2.71 (1.09, 4.33)	3.46 (2.23, 4.68)	4.28 (2.81, 5.76)	Tramadol	3.12 (1.92, 4.33)
Tramadol + acetaminophen	0.76 (−0.29, 1.81)	−0.07 (−0.8, 0.66)	0.59 (−0.28, 1.45)	−2.1 (−3.55, −0.66)	−0.22 (−0.71, 0.27)	0.46 (−0.39, 1.31)	−0.41 (−1.7, 0.87)	0.33 (−0.39, 1.05)	1.16 (0.06, 2.25)	−3.12 (−4.33, −1.92)	Tramadol + acetaminophen
Dizziness											
	Buprenorphine	Codeine + acetaminophen	Fentanyl	Fentanyl + tramadol	Hydrocodone + acetaminophen	Morphine	Oxycodone	Oxycodone + acetaminophen	Sufentanil	Tramadol	
Buprenorphine	Buprenorphine	1.65 (0.45, 6.36)	1.01 (0.24, 4.18)	1.98 (0.18, 21.56)	2.02 (0.59, 7.26)	1.57 (0.66, 3.98)	1.47 (0.49, 4.64)	2.17 (0.61, 8.13)	1.23 (0.45, 3.52)	2.73 (0.41, 20.47)	
Codeine + acetaminophen	0.61 (0.16, 2.23)	Codeine + acetaminophen	0.62 (0.19, 1.82)	1.19 (0.14, 9.96)	1.22 (0.79, 1.92)	0.96 (0.35, 2.52)	0.89 (0.27, 2.91)	1.32 (0.86, 2.05)	0.75 (0.24, 2.2)	1.64 (0.34, 8.98)	
Fentanyl	0.99 (0.24, 4.24)	1.62 (0.55, 5.34)	Fentanyl	1.95 (0.19, 20.32)	1.98 (0.70, 6.31)	1.54 (0.52, 4.80)	1.46 (0.40, 5.67)	2.13 (0.79, 6.40)	1.21 (0.36, 4.34)	2.69 (0.44, 18.89)	
Fentanyl + tramadol	0.51 (0.05, 5.49)	0.84 (0.1, 7.13)	0.51 (0.05, 5.17)	Fentanyl + tramadol	1.03 (0.13, 8.35)	0.80 (0.09, 7.40)	0.75 (0.07, 7.55)	1.11 (0.13, 9.32)	0.63 (0.06, 6.07)	1.37 (0.37, 5.90)	
Hydrocodone + acetaminophen	0.5 (0.14, 1.7)	0.82 (0.52, 1.28)	0.5 (0.16, 1.42)	0.97 (0.12, 7.84)	Hydrocodone + acetaminophen	0.78 (0.32, 1.84)	0.73 (0.24, 2.22)	1.08 (0.72, 1.61)	0.61 (0.22, 1.66)	1.40 (0.29, 7.10)	
Morphine	0.64 (0.25, 1.51)	1.05 (0.4, 2.85)	0.64 (0.2, 1.93)	1.25 (0.14, 11.44)	1.28 (0.54, 3.18)	Morphine	0.93 (0.46, 1.86)	1.38 (0.54, 3.61)	0.78 (0.46, 1.28)	1.72 (0.32, 11.00)	
Oxycodone	0.68 (0.22, 2.06)	1.12 (0.34, 3.73)	0.69 (0.18, 2.51)	1.34 (0.13, 13.58)	1.37 (0.45, 4.25)	1.07 (0.54, 2.13)	Oxycodone	1.47 (0.47, 4.79)	0.84 (0.35, 1.95)	1.84 (0.30, 12.78)	
Oxycodone + acetaminophen	0.46 (0.12, 1.63)	0.76 (0.49, 1.17)	0.47 (0.15, 1.29)	0.9 (0.11, 7.57)	0.93 (0.62, 1.38)	0.73 (0.28, 1.84)	0.68 (0.21, 2.14)	Oxycodone + acetaminophen	0.57 (0.19, 1.62)	1.24 (0.26, 6.70)	
Sufentanil	0.82 (0.28, 2.21)	1.34 (0.45, 4.1)	0.82 (0.23, 2.77)	1.6 (0.16, 15.65)	1.64 (0.6, 4.65)	1.28 (0.78, 2.14)	1.2 (0.51, 2.82)	1.77 (0.62, 5.25)	Sufentanil	2.21 (0.38, 14.46)	
Tramadol	0.37 (0.05, 2.45)	0.61 (0.11, 2.96)	0.37 (0.05, 2.28)	0.73 (0.17, 2.66)	0.75 (0.14, 3.38)	0.58 (0.09, 3.12)	0.54 (0.08, 3.35)	0.81 (0.15, 3.81)	0.45 (0.07, 2.63)	Tramadol	
Hypotension											
	Buprenorphine	Morphine	Oxycodone	Sufentanil							
Buprenorphine	Buprenorphine	5.01 (1.31, 35.76)	10.17 (1.72, 99.93)	5.44 (0.11, 301.65)							
Morphine	0.20 (0.03, 0.77)	Morphine	1.95 (0.67, 6.9)	1.03 (0.03, 37.05)							
Oxycodone	0.10 (0.01, 0.58)	0.51 (0.14, 1.49)	Oxycodone	0.51 (0.01, 21.71)							
Sufentanil	0.18 (0, 8.96)	0.97 (0.03, 37.16)	1.95 (0.05, 88.92)	Sufentanil							
Pruritus											
	Butorphanol	Codeine + acetaminophen	Fentanyl	Hydrocodone + acetaminophen	Morphine	Oxycodone + acetaminophen					
Butorphanol	Butorphanol	2.3 (0.04, 132.5)	1.14 (0.02, 75.4)	2.12 (0.05, 87.24)	1.11 (0.03, 37.85)	2.81 (0.07, 115.37)					
Codeine + acetaminophen	0.43 (0.01, 28.16)	Codeine + acetaminophen	0.49 (0.03, 10.64)	0.89 (0.14, 7.84)	0.47 (0.07, 4.25)	1.19 (0.20, 10.12)					
Fentanyl	0.88 (0.01, 59.66)	2.06 (0.09, 38.7)	Fentanyl	1.87 (0.16, 21.33)	0.98 (0.10, 9.20)	2.51 (0.22, 28.27)					
Hydrocodone + acetaminophen	0.47 (0.01, 19.68)	1.11 (0.13, 7.28)	0.53 (0.05, 6.08)	Hydrocodone + acetaminophen	0.53 (0.19, 1.37)	1.32 (0.39, 4.8)					
Morphine	0.9 (0.03, 33.76)	2.14 (0.24, 14.12)	1.02 (0.11, 9.5)	1.9 (0.73, 5.42)	Morphine	2.51 (0.98, 7.24)					
Oxycodone + acetaminophen	0.36 (0.01, 14.73)	0.84 (0.1, 5.05)	0.4 (0.04, 4.56)	0.76 (0.21, 2.6)	0.4 (0.13, 1.02)	Oxycodone + acetaminophen					
Sedation											
	Butorphanol	Fentanyl	Hydrocodone + acetaminophen	Morphine							
Butorphanol	Butorphanol	2.33 (0.41, 19.88)	0.23 (0.05, 0.81)	0.83 (0.39, 1.71)							
Fentanyl	0.43 (0.05, 2.46)	Fentanyl	0.10 (0.01, 0.66)	0.36 (0.05, 1.71)							
Hydrocodone + acetaminophen	4.38 (1.23, 19.25)	10.43 (1.51, 107.93)	Hydrocodone + acetaminophen	3.59 (1.30, 12.96)							
Morphine	1.2 (0.59, 2.57)	2.78 (0.58, 21)	0.28 (0.08, 0.77)	Morphine							
Rescue analgesia											
	Butorphanol	Codeine + acetaminophen	Fentanyl	Hydrocodone + acetaminophen	Morphine	Oxycodone	Oxycodone + acetaminophen	Sufentanil			
Butorphanol	Butorphanol	7.30 (0.20, 286.06)	1.49 (0.11, 21.64)	7.14 (0.21, 267.85)	1.49 (0.13, 17.84)	0.11 (0, 4.58)	5.48 (0.20, 163.67)	1.55 (0.07, 36.72)			
Codeine + acetaminophen	0.14 (0, 4.91)	Codeine + acetaminophen	0.21 (0.02, 2.39)	0.97 (0.26, 3.74)	0.21 (0.01, 2.97)	0.02 (0, 0.71)	0.75 (0.19, 2.84)	0.21 (0.01, 5.91)			
Fentanyl	0.67 (0.05, 9.06)	4.85 (0.42, 60.27)	Fentanyl	4.72 (0.44, 54.58)	1.00 (0.36, 2.78)	0.08 (0, 1.37)	3.64 (0.46, 30.44)	1.04 (0.11, 9.6)			
Hydrocodone + acetaminophen	0.14 (0, 4.79)	1.03 (0.27, 3.91)	0.21 (0.02, 2.26)	Hydrocodone + acetaminophen	0.21 (0.01, 2.79)	0.02 (0, 0.69)	0.77 (0.23, 2.45)	0.22 (0.01, 5.61)			
Morphine	0.67 (0.06, 7.48)	4.84 (0.34, 73.01)	1 (0.36, 2.77)	4.72 (0.36, 67.24)	Morphine	0.08 (0, 1.13)	3.64 (0.36, 38.61)	1.03 (0.14, 7.61)			
Oxycodone	8.84 (0.22, 701.19)	64.75 (1.41, 5,981.2)	12.76 (0.73, 595.27)	62.6 (1.44, 5,654.85)	12.66 (0.88, 525.94)	Oxycodone	48.2 (1.35, 3,694.76)	13.41 (0.47, 884.32)			
Oxycodone + acetaminophen	0.18 (0.01, 5.11)	1.33 (0.35, 5.19)	0.27 (0.03, 2.17)	1.29 (0.41, 4.32)	0.27 (0.03, 2.76)	0.02 (0, 0.74)	Oxycodone + acetaminophen	0.28 (0.01, 5.99)			
Sufentanil	0.65 (0.03, 14.39)	4.69 (0.17, 135.53)	0.97 (0.1, 8.94)	4.58 (0.18, 126.46)	0.97 (0.13, 7.02)	0.07 (0, 2.12)	3.53 (0.17, 76.58)	Sufentanil			

**TABLE 2:** Rank probability of various opioid drugs for different outcomes in trauma patients from the emergency department.

Pain relief											
	[1]	[2]	[3]	[4]	[5]	[6]	[7]	[8]	[9]	[10]	[11]
Buprenorphine	0.184,615	0.4854	0.122,225	0.090675	0.060375	0.030605	0.01466	0.0098	0.0016	0.000045	0
Codeine + acetaminophen	0.0015	0.009135	0.017495	0.02355	0.04865	0.25388	0.28982	0.245,865	0.1092	0.000905	0
Fentanyl	0.01484	0.24658	0.53248	0.137,775	0.04579	0.01567	0.00569	0.00111	0.000065	0	0
Fentanyl + tramadol	0.000015	0.00009	0.0001	0.000095	0.000275	0.00072	0.001115	0.00204	0.03072	0.958,775	0.006055
Hydrocodone + acetaminophen	0.000065	0.000645	0.00299	0.005705	0.01121	0.0532	0.247,265	0.441,565	0.23657	0.000785	0
Morphine	0.00011	0.009205	0.140,525	0.53251	0.20625	0.07292	0.027295	0.0107	0.000485	0	0
Oxycodone	0.00189	0.00913	0.01267	0.01353	0.04742	0.121,045	0.11002	0.106,925	0.54462	0.032315	0.000435
Oxycodone + acetaminophen	0.00517	0.047685	0.100,135	0.13898	0.48651	0.175,805	0.037645	0.007465	0.000605	0	0
Sufentanil	0.782,885	0.157,395	0.02836	0.015595	0.008865	0.003995	0.00167	0.0011	0.000125	0.00001	0
Tramadol	0	0	0	0	0	0	0	0	0	0.00649	0.99351
Tramadol + acetaminophen	0.00891	0.034735	0.04302	0.041585	0.084655	0.27216	0.26482	0.17343	0.07601	0.000675	0
Dizziness											
	[1]	[2]	[3]	[4]	[5]	[6]	[7]	[8]	[9]	[10]	
Buprenorphine	0.0219	0.022985	0.033885	0.03884	0.04797	0.06998	0.09314	0.140,565	0.218,775	0.31196	
Codeine + acetaminophen	0.01491	0.04855	0.110,295	0.148,055	0.20381	0.13161	0.11988	0.10992	0.081895	0.031075	
Fentanyl	0.016435	0.019295	0.03108	0.039825	0.058265	0.09294	0.09578	0.135,375	0.20135	0.309,655	
Fentanyl + tramadol	0.22141	0.19782	0.051755	0.051315	0.061825	0.04732	0.0478	0.06191	0.087885	0.17096	
Hydrocodone + acetaminophen	0.065825	0.15156	0.228,195	0.222,365	0.143,975	0.088155	0.057655	0.03025	0.01044	0.00158	
Morphine	0.034135	0.06994	0.0999	0.12652	0.15528	0.212,835	0.19305	0.089555	0.01766	0.001125	
Oxycodone	0.074775	0.064735	0.08971	0.083505	0.099115	0.13633	0.14052	0.139,735	0.11354	0.058035	
Oxycodone + acetaminophen	0.172,675	0.182,455	0.24264	0.16359	0.097475	0.06474	0.044445	0.024085	0.00705	0.000845	
Sufentanil	0.015045	0.02495	0.04295	0.05576	0.075835	0.111,555	0.16098	0.21552	0.20171	0.095695	
Tramadol	0.36289	0.21771	0.06959	0.070225	0.05645	0.044535	0.04675	0.053085	0.059695	0.01907	
Hypotension											
	[1]	[2]	[3]	[4]							
Buprenorphine	0.000965	0.005255	0.17737	0.81641							
Morphine	0.055365	0.490,855	0.4502	0.00358							
Oxycodone	0.60317	0.331,175	0.06315	0.002505							
Sufentanil	0.3405	0.172,715	0.30928	0.177,505							
Pruritus											
	[1]	[2]	[3]	[4]	[5]	[6]					
Butorphanol	0.195,345	0.07988	0.080075	0.099445	0.13968	0.405,575					
Codeine + acetaminophen	0.28073	0.206,175	0.16505	0.12695	0.11779	0.103,305					
Fentanyl	0.12446	0.09822	0.11019	0.146,285	0.22378	0.297,065					
Hydrocodone + acetaminophen	0.1206	0.25374	0.317,805	0.206,425	0.07984	0.02159					
Morphine	0.00089	0.013145	0.08939	0.317,905	0.411,165	0.167,505					
Oxycodone + acetaminophen	0.277,975	0.34884	0.23749	0.10299	0.027745	0.00496					
Sedation											
	[1]	[2]	[3]	[4]							
Butorphanol	0.15568	0.552,435	0.282,525	0.00936							
Fentanyl	0.81256	0.09776	0.08179	0.00789							
Hydrocodone + acetaminophen	0.000345	0.003225	0.017185	0.979,245							
Morphine	0.031415	0.34658	0.6185	0.003505							
Rescue analgesia											
	[1]	[2]	[3]	[4]	[5]	[6]	[7]	[8]			
Butorphanol	0.063925	0.03708	0.03887	0.14465	0.099405	0.130,805	0.38311	0.102,155			
Codeine + acetaminophen	0.38273	0.281,815	0.18577	0.05997	0.03513	0.028405	0.02102	0.00516			
Fentanyl	0.01002	0.02128	0.037575	0.237,995	0.277,975	0.259,115	0.145,345	0.010695			
Hydrocodone + acetaminophen	0.332,535	0.333,135	0.190,665	0.05953	0.034685	0.027635	0.01773	0.004085			
Morphine	0.013735	0.035465	0.0459	0.156,735	0.34814	0.30587	0.09172	0.002435			
Oxycodone	0.003405	0.0033	0.00408	0.01103	0.01432	0.024875	0.10264	0.83635			
Oxycodone + acetaminophen	0.110,355	0.239,315	0.443,145	0.1025	0.049195	0.0339	0.01845	0.00314			
Sufentanil	0.083295	0.04861	0.053995	0.22759	0.14115	0.189,395	0.219,985	0.03598			

### Network meta-analysis for adverse events

#### Dizziness

The incidence of dizziness after the following 10 opioid medications was explored by 11 studies with 1,265 patients: buprenorphine, codeine + acetaminophen, fentanyl, fentanyl + tramadol, hydrocodone + acetaminophen, morphine, oxycodone, oxycodone + acetaminophen, sufentanil, and tramadol. The direct comparison of morphine and fentanyl was conducted in more studies. Morphine and hydrocodone + acetaminophen were used in more patients (Supplementary Figure S1).

No significant difference was found between the investigated opioid medications, according to the forest plot (Supplementary Figure S2) and league table (Table 1). Based on the rank probability, buprenorphine (31.20%), fentanyl (20.14%) and sufentanil (21.55%) were least likely to cause dizziness (Table 2).

#### Hypotension

Three studies with 293 patients provided data on the influence of four analgesics on hypotension: morphine, sufentanil, oxycodone, and buprenorphine. Morphine was the most commonly studied analgesics. Morphine was directly compared with other three drugs (Supplementary Figure S1).

The forest plot demonstrated that patients using buprenorphine had a significantly decreased incidence of hypotension in contrast to those using morphine (pooled RR = 0.20, 95%CrI: 0.031, 0.79). As shown in the league table, the incidence of hypotension was significantly higher after morphine administration *versus* buprenorphine administration (pooled RR = 5.01, 95%CrI: 1.31, 35.76). Oxycodone was associated with a significantly increased incidence of hypotension compared with buprenorphine (pooled RR = 10.17, 95%CrI: 1.72, 99.93) (Table 1). The rank probability indicated that the top three analgesic medications which were least likely to cause hypotension were buprenorphine (81.64%), morphine (45.02%) and sufentanil (17.27%) (Table 2).

#### Pruritus

The impact of opioid medications on pruritus was evaluated in 6 studies of 1,048 patients, and 6 medications were involved: buprenorphine, codeine + acetaminophen, fentanyl, hydrocodone + acetaminophen, morphine, and oxycodone + acetaminophen. Morphine was directly compared with fentanyl in more studies, and more patients were administrated morphine and oxycodone + acetaminophen (Supplementary Figure S1).

The forest plot exhibited that patients using oxycodone + acetaminophen had a significantly greater incidence of pruritus than those using morphine (pooled RR = 4.10, 95%CrI: 1.30, 18.00) (Supplementary Figure S2). In the league table, comparable incidences of pruritus were shown after using the 6 medications (Table 1). As suggested by the rank probability, butorphanol (40.56%), morphine (41.11%) and fentanyl (14.63%) were least likely to cause pruritus (Table 2).

#### Sedation

Four studies of 368 patients assessed the effect of butorphanol, fentanyl, hydrocodone + acetaminophen, and morphine on sedation. Morphine was the most commonly studied opioid, and it was directly compared with the other three analgesics (Supplementary Figure S1).

Patients using hydrocodone + acetaminophen had a significantly lower incidence of sedation compared with those using morphine, as displayed by the direct comparison result of the forest plot (pooled RR = 0.28, 95%CrI: 0.077, 0.77). The league table found that morphine was associated with a significantly elevated incidence of sedation than hydrocodone + acetaminophen (Table 1). According to the rank probability, the top three medications which were least likely to cause sedation were hydrocodone + acetaminophen (97.92%), morphine (61.85%) and butorphanol (55.24%) (Table 2).

### Network meta-analysis for rescue analgesia

Data on rescue analgesia were provided by 11 studies with 1,755 patients, involving 8 opioid medications: butorphanol, codeine + acetaminophen, fentanyl, hydrocodone + acetaminophen, morphine, oxycodone, oxycodone + acetaminophen, and sufentanil. More studies investigated the direct comparison between morphine and fentanyl, and more patients used these two opioids (Supplementary Figure S1).

The forest plot demonstrated no significant difference between the presented medications (Supplementary Figure S2). According to the league table, compared with patients receiving codeine + acetaminophen (pooled RR = 0.02, 95%CrI: 0.00, 0.71) or hydrocodone + acetaminophe (pooled RR = 0.02, 95%CrI: 0.00, 0.69), patients receiving oxycodone had a significantly reduced need for rescue analgesia. The need for rescue analgesia following oxycodone + acetaminophen was significantly greater than that following oxycodone (pooled RR = 48.20, 95%CrI: 1.35, 3,694.76) (Table 1). The rank probability showed that patients who received oxycodone (83.64%), butorphanol (38.31%) and fentanyl (25.91%) were least likely to need rescue analgesia (Table 2). Soysal et al. (Soysal et al., 2004) reported that patients receiving meperidine + midazolam needed rescue analgesia more frequently than those receiving fentanyl + midazolam (21% vs. 9%).

## Discussion

In the current network meta-analysis, the efficacy of opioid medications for traumatic pain in the emergency department was first systematically evaluated as regards pain relief, adverse events and rescue analgesia. We found that sufentanil, buprenorphine and fentanyl may be more effective in relieving pain, and may relate to a lower incidence of dizziness, hypotension and pruritus, and need for rescue analgesia among opioid medications for trauma patients, suggesting that sufentanil, buprenorphine and fentanyl may have advantages over other opioid medications in treating traumatic pain, which might be chosen as opioid analgesics in the emergency department.

A network meta-analysis was recently performed by Yin et al. (Yin et al., 2021) to assess the efficacy of various analgesics, including opioids, in traumatic musculoskeletal pain. It was found that NSAIDs exhibited the greatest overall efficacy, and opioids were the optimal medications for pain intensity at 60 min. This network meta-analysis focused on the comparison of opioids for traumatic pain in the emergency department, and illustrated that patients receiving sufentanil, buprenorphine and fentanyl may have better pain relief, and a lower incidence of dizziness, hypotension and pruritus, and need for rescue analgesia than those receiving other opioid medications. As for pain relief, Lemoel et al. (Lemoel et al., 2019) reported that intranasal sufentanil in the emergency setting significantly raised the number of patients with severe pain achieving pain relief within 30 min. Sufentanil is a more potent opioid drug, which is well known to emergency physicians, and this inexpensive lipophilic agent has fast onset of action (within 20 min) and short half-life (15–20 min) (Stephen et al., 2012; Corrigan et al., 2015). A previous observational study showed effective pain relief after intranasal sufentanil administration, with low incidences of dizziness, vomiting and hypoxia and without presence of hypotension and apnea in patients with acute traumatic extremity injuries (Steenblik et al., 2012). Sufentanil also exhibited its efficacy in managing severe pain for individuals with distal extremity injury, without serious adverse events (Stephen et al., 2012). Sin et al. (Sin et al., 2019) found similar impacts of sufentanil and morphine on acute pain reduction in the emergency department, while our findings favored sufentanil over morphine. This difference may be attributed to the relatively small sample size and different study population of the former. Additionally, Sufentanil (26,716) has a higher therapeutic index than fentanyl (277) and morphine (71) according to preclinical studies, and it exhibits high bioavailability (Helmers et al., 1989; Mather, 1995). Hence, sufentanil can exert greater analgesic effects over longer time compared with the same dose of fentanyl or morphine, and can lower the incidence of respiratory depression and adverse events (Xu et al., 2020). Given the significant harm that opioid abuse poses to public health (Volkow and McLellan, 2016), greater potency of sufentanil *versus* fentanyl and morphine may lead to accidental overdose of sufentanil (Deeks, 2019). Sufentanil is limited to use under medical supervision, which may alleviate this concern. Based on the above, sufentanil may be an attractive analgesic drug for patients with traumatic pain in the emergency department. Buprenorphine, an opioid receptor agonist-antagonist, was illustrated to be a more potent opioid than others, such as morphine, fentanyl and tramadol in pain treatment, with high safety and longer action time (Sporer, 2004; Leffler et al., 2012). Murray et al. (Murray et al., 2018) showed that buprenorphine provided longer analgesia time than morphine for pediatric acute pain. According to prior evidence, buprenorphine played an effective role in analgesia for both adult and pediatric patients undergoing surgery with fewer side effects (Giron et al., 2022). Recently, buprenorphine treatment has become more and more common in the emergency setting (Pourmand et al., 2021). Fentanyl has been used in anesthesia since 1960. Different routes of administration make fentanyl a good choice in emergencies, and fentanyl has been applied for acute pain relief (Furyk et al., 2009). It has onset time of less than 60 s, a half-life of 90 min, and a duration of about 30–60 min, with minor cardiovascular impacts (Stanley, 2014). This network meta-analysis combined direct and indirect evidence for opioid efficacy assessment, and sufentanil, buprenorphine and fentanyl may rank the top three for pain relief, which might be considered by physicians in the management of traumatic pain.

Concerning adverse events, the incidence of dizziness after sufentanil, buprenorphine and fentanyl may be lower than that after the other opioid medications based on the result of the rank probability, although no significant difference were shown. For hypotension, only four opioids were evaluated, and buprenorphine may be associated with the lowest incidence of hypotension, while fentanyl was not investigated in the qualified studies. For pruritus and sedation, sufentanil and buprenorphine were not assessed by the included studies, and patients using fentanyl may have the third lowest incidence of pruritus but an unfavorable incidence of sedation. More studies are needed in the future to verify the role of different opioids in consideration of adverse events. Of note, the proportion of patients who required rescue analgesia may be relatively low after receiving fentanyl, and relatively more patients may require rescue analgesia after sufentanil treatment, which necessitated large-scale studies for corroboration.

Based on our findings from this comprehensive assessment with 20 studies of 3,040 patients, sufentanil, buprenorphine and fentanyl may provide favorable effects on traumatic pain relief, and may be associated with a lower incidence of dizziness, hypotension and pruritus, and need for rescue analgesia, which might serve as a reference in choosing opioid medications for traumatic pain management in the emergency department. Some limitations should be noted in interpreting the findings. Firstly, the dosage of same medications varied across different studies, which precipitated increased study heterogeneity. Secondly, equipotent doses were not studied given the clinical limitations of care of the trauma research subjects enrolled in many of the studies, therefore higher and lower doses were titrated to clinical effect. If the doses compared were not equipotent, this may affect safety and efficacy in traumatic pain. For example, higher doses might provide more pain relief. Besides, the small sample size of the included studies may affect the stability of our results. Thirdly, different administration routes of the same medication may affect the analgesic effect, and subgroup analysis based on administration routes could not be achieved due to limited eligible studies. As shown in Supplementary Figure S3, opioids with similar routes of administration cannot form a complete network plot. For instance, as regards the outcome pain relief, IV tramadol cannot form a network plot with IV fentanyl and IV morphine, so it cannot be compared with IV fentanyl and IV morphine. Likewise, oral oxycodone cannot be compared with other oral opioid medications. Besides, only buprenorphine is administered sublingually, so there is no comparison between sublingual (SL) buprenorphine and other opioid medications with similar routes of administration. The outcomes adverse events and rescue analgesia had similar situations to the outcome pain relief. More studies are required in the future to compare similar routes of opioid administration. Language bias also existed in this analysis since merely English literature was included.

## Conclusion

For trauma patients with musculoskeletal or trauma-related pain in the emergency department, sufentanil, buprenorphine and fentanyl may provide favorable effects on pain relief, and may be associated with a lower incidence of dizziness, hypotension and pruritus, and need for rescue analgesia among opioid medications. Confirmation of these finding requires further studies.

## Data Availability

The raw data supporting the conclusions of this article will be made available by the authors, without undue reservation.

## Supplementary material

SUPPLEMENTARY FIGURE S1 Network plots of various opioid drugs for different outcomes in trauma patients from the emergency department. **(A)** pain relief; **(B)** dizziness; **(C)** hypotension; **(D)** pruritus; **(E)** sedation; **(F)** rescue analgesia.

SUPPLEMENTARY FIGURE S2 Forest plots of various opioid drugs for different outcomes in trauma patients from the emergency department. **(A)** pain relief; **(B)** dizziness; **(C)** pruritus; **(D)** rescue analgesia.

SUPPLEMENTARY FIGURE S3 Network plots of opioid drugs with various routes of administration for pain relief in trauma patients from the emergency department. IV, intravenous; INH, inhalation; SL, sublingual.

## References

[B1] Abdolrazaghnejad A. Banaie M. Tavakoli N. Safdari M. Rajabpour-Sanati A. (2018). Pain management in the emergency department: A review article on options and methods. Adv. J. Emerg. Med. 2 (4), e45. 10.22114/AJEM.v0i0.93 31172108 PMC6548151

[B2] Abu-Snieneh H. M. Alsharari A. F. Abuadas F. H. Alqahtani M. E. (2022). Effectiveness of pain management among trauma patients in the emergency department, a systematic review. Int. Emerg. Nurs. 62, 101158. 10.1016/j.ienj.2022.101158 35364460

[B3] Ahmadi A. Bazargan-Hejazi S. Heidari Zadie Z. Euasobhon P. Ketumarn P. Karbasfrushan A. (2016). Pain management in trauma: A review study. J. Inj. Violence Res. 8 (2), 89–98. 10.5249/jivr.v8i2.707 27414816 PMC4967367

[B4] Bijur P. E. Friedman B. W. Irizarry E. Chang A. K. Gallagher E. J. (2021). A randomized trial comparing the efficacy of five oral analgesics for treatment of acute musculoskeletal extremity pain in the emergency department. Ann. Emerg. Med. 77 (3), 345–356. 10.1016/j.annemergmed.2020.10.004 33358232

[B5] Blancher M. Maignan M. Clapé C. Quesada J. L. Collomb-Muret R. Albasini F. (2019). Intranasal sufentanil versus intravenous morphine for acute severe trauma pain: A double-blind randomized non-inferiority study. PLoS Med. 16 (7), e1002849. 10.1371/journal.pmed.1002849 31310600 PMC6634380

[B6] Bounes V. Barthelemy R. Diez O. Charpentier S. Montastruc J. L. Ducasse J. L. (2010). Sufentanil is not superior to morphine for the treatment of acute traumatic pain in an emergency setting: A randomized, double-blind, out-of-hospital trial. Ann. Emerg. Med. 56 (5), 509–516. 10.1016/j.annemergmed.2010.03.020 20382445

[B7] Chang A. K. Bijur P. E. Esses D. Barnaby D. P. Baer J. (2017). Effect of a single dose of oral opioid and nonopioid analgesics on acute extremity pain in the emergency department: A randomized clinical trial. Jama 318 (17), 1661–1667. 10.1001/jama.2017.16190 29114833 PMC5818795

[B8] Chew K. S. Shaharudin A. H. (2017). An open-label randomised controlled trial on the efficacy of adding intranasal fentanyl to intravenous tramadol in patients with moderate to severe pain following acute musculoskeletal injuries. Singap. Med. J. 58 (10), 601–605. 10.11622/smedj.2016096 PMC5651506 27193080

[B9] Corrigan M. Wilson S. S. Hampton J. (2015). Safety and efficacy of intranasally administered medications in the emergency department and prehospital settings. Am. J. Health Syst. Pharm. 72 (18), 1544–1554. 10.2146/ajhp140630 26346210

[B10] Deeks E. D. (2019). Sufentanil 30 µg sublingual tablet: A review in acute pain. Clin. drug Investig. 39 (4), 411–418. 10.1007/s40261-019-00772-x 30887417

[B11] Devall A. J. Papadopoulou A. Podesek M. Haas D. M. Price M. J. Coomarasamy A. (2021). Progestogens for preventing miscarriage: A network meta-analysis. Cochrane database Syst. Rev. 4 (4), CD013792. 10.1002/14651858.CD013792.pub2 33872382 PMC8406671

[B12] Dijkstra B. M. Berben S. A. van Dongen R. T. Schoonhoven L. (2014). Review on pharmacological pain management in trauma patients in (pre-hospital) emergency medicine in The Netherlands. Eur. J. Pain 18 (1), 3–19. 10.1002/j.1532-2149.2013.00337.x 23737462

[B13] Dißmann P. D. Maignan M. Cloves P. D. Gutierrez Parres B. Dickerson S. Eberhardt A. (2018). A review of the burden of trauma pain in emergency settings in europe. Pain Ther. 7 (2), 179–192. 10.1007/s40122-018-0101-1 29860585 PMC6251834

[B14] Eizadi P. Jalili M. Dehpour A. (2018). Oral oxycodone compared with intravenous morphine sulfate for pain management of isolated limb trauma; a randomized clinical trial. Emerg. (Tehran) 6 (1), e59. 30584575 PMC6289158

[B15] Furyk J. S. Grabowski W. J. Black L. H. (2009). Nebulized fentanyl versus intravenous morphine in children with suspected limb fractures in the emergency department: A randomized controlled trial. Emerg. Med. Australas. 21 (3), 203–209. 10.1111/j.1742-6723.2009.01183.x 19527280

[B16] Giron S. E. Lai G. Griffis C. A. Zhang S. J. (2022). Demystifying buprenorphine with current evidence-based practice in acute and chronic pain management. Aana J. 90 (3), 225–233. 35604865

[B17] Guyatt G. H. Oxman A. D. Vist G. Kunz R. Brozek J. Alonso-Coello P. (2011). GRADE guidelines: 4. Rating the quality of evidence--study limitations (risk of bias). J. Clin. Epidemiol. 64 (4), 407–415. 10.1016/j.jclinepi.2010.07.017 21247734

[B18] Helmers J. H. Noorduin H. Van Peer A. Van Leeuwen L. Zuurmond W. W. (1989). Comparison of intravenous and intranasal sufentanil absorption and sedation. Can. J. Anaesth. 36 (5), 494–497. 10.1007/bf03005373 2529048

[B19] Hewitt D. J. Todd K. H. Xiang J. Jordan D. M. Rosenthal N. R. Investigators C.-S. (2007). Tramadol/acetaminophen or hydrocodone/acetaminophen for the treatment of ankle sprain: A randomized, placebo-controlled trial. Ann. Emerg. Med. 49 (4), 468–480. 10.1016/j.annemergmed.2006.08.030 17113683

[B20] Higgins J. P. Altman D. G. Gøtzsche P. C. Jüni P. Moher D. Oxman A. D. (2011). The Cochrane Collaboration's tool for assessing risk of bias in randomised trials. Bmj 343, d5928. 10.1136/bmj.d5928 22008217 PMC3196245

[B21] Hutton B. Salanti G. Caldwell D. M. Chaimani A. Schmid C. H. Cameron C. (2015). The PRISMA extension statement for reporting of systematic reviews incorporating network meta-analyses of health care interventions: Checklist and explanations. Ann. Intern Med. 162 (11), 777–784. 10.7326/m14-2385 26030634

[B22] Jalili M. Fathi M. Moradi-Lakeh M. Zehtabchi S. (2012). Sublingual buprenorphine in acute pain management: A double-blind randomized clinical trial. Ann. Emerg. Med. 59 (4), 276–280. 10.1016/j.annemergmed.2011.10.021 22115823

[B23] Kariman H. Majidi A. Amini A. Dolatabadi A. A. Derakhshanfar H. Hatamabadi H. (2011). Nitrous oxide/oxygen compared with fentanyl in reducing pain among adults with isolated extremity trauma: A randomized trial. Emerg. Med. Australas. 23 (6), 761–768. 10.1111/j.1742-6723.2011.01447.x 22151676

[B24] Kim H. S. Heard K. J. Heard S. Hoppe J. A. (2016). Opioid prescription fill rates after emergency department discharge. Am. J. Health Syst. Pharm. 73 (12), 902–907. 10.2146/ajhp150528 27261241

[B25] Leffler A. Frank G. Kistner K. Niedermirtl F. Koppert W. Reeh P. W. (2012). Local anesthetic-like inhibition of voltage-gated Na(+) channels by the partial μ-opioid receptor agonist buprenorphine. Anesthesiology 116 (6), 1335–1346. 10.1097/ALN.0b013e3182557917 22504149

[B26] Lemoel F. Contenti J. Cibiera C. Rapp J. Occelli C. Levraut J. (2019). Intranasal sufentanil given in the emergency department triage zone for severe acute traumatic pain: A randomized double-blind controlled trial. Intern Emerg. Med. 14 (4), 571–579. 10.1007/s11739-018-02014-y 30600526

[B27] Mahil S. K. Ezejimofor M. C. Exton L. S. Manounah L. Burden A. D. Coates L. C. (2020). Comparing the efficacy and tolerability of biologic therapies in psoriasis: An updated network meta-analysis. Br. J. dermatology 183 (4), 638–649. 10.1111/bjd.19325 32562551

[B28] Marco C. A. Plewa M. C. Buderer N. Black C. Roberts A. (2005). Comparison of oxycodone and hydrocodone for the treatment of acute pain associated with fractures: A double-blind, randomized, controlled trial. Acad. Emerg. Med. 12 (4), 282–288. 10.1197/j.aem.2004.12.005 15805317

[B29] Mather L. E. (1995). Opioids: A pharmacologist's delight. Clin. Exp. Pharmacol. physiology 22 (11), 833–836. 10.1111/j.1440-1681.1995.tb01945.x 8593739

[B30] Miller P. L. Ernst A. A. (2004). Sex differences in analgesia: A randomized trial of mu versus kappa opioid agonists. South. Med. J. 97 (1), 35–41. 10.1097/01.Smj.0000085743.68121.A9 14746420

[B31] Murray N. Malla U. Vlok R. Scott A. Chua O. Melhuish T. (2018). Buprenorphine versus morphine in paediatric acute pain: A systematic review and meta-analysis. Crit. Care Res. Pract. 2018, 3792043. 10.1155/2018/3792043 30159170 PMC6109565

[B32] Pan Z. Q. Qi Y. J. Wen Y. X. Chen L. B. (2018). Intravenous morphine titration vs. oral hydrocodone/acetaminophen for adults with lower extremity displaced fracture in an emergency department setting: A randomized controlled trial. Exp. Ther. Med. 16 (4), 3674–3679. 10.3892/etm.2018.6606 30233725 PMC6143845

[B33] Papakonstantinou T. Nikolakopoulou A. Egger M. Salanti G. (2020). In network meta-analysis, most of the information comes from indirect evidence: Empirical study. J. Clin. Epidemiol. 124, 42–49. 10.1016/j.jclinepi.2020.04.009 32302680

[B34] Porter K. M. Siddiqui M. K. Sharma I. Dickerson S. Eberhardt A. (2018). Management of trauma pain in the emergency setting: Low-dose methoxyflurane or nitrous oxide? A systematic review and indirect treatment comparison. J. Pain Res. 11, 11–21. 10.2147/jpr.S150600 29302193 PMC5741984

[B35] Pourmand A. Beisenova K. Shukur N. Tebo C. Mortimer N. Mazer-Amirshahi M. (2021). A practical review of buprenorphine utilization for the emergency physician in the era of decreased prescribing restrictions. Am. J. Emerg. Med. 48, 316–322. 10.1016/j.ajem.2021.06.065 34274576

[B36] Rouse B. Chaimani A. Li T. (2017). Network meta-analysis: An introduction for clinicians. Intern. Emerg. Med. 12 (1), 103–111. 10.1007/s11739-016-1583-7 27913917 PMC5247317

[B37] Shear M. L. Adler J. N. Shewakramani S. Ilgen J. Soremekun O. A. Nelson S. (2010). Transbuccal fentanyl for rapid relief of orthopedic pain in the ED. Am. J. Emerg. Med. 28 (8), 847–852. 10.1016/j.ajem.2009.04.011 20887903

[B38] Shervin F. Said S. Mohammad-Taghi T. Shahram B. H. Mona A. Hamed B. (2014). Nebulized fentanyl vs intravenous morphine for ED patients with acute limb pain: A randomized clinical trial. Am. J. Emerg. Med. 32 (9), 1011–1015. 10.1016/j.ajem.2014.05.051 25027194

[B39] Sin B. Jeffrey I. Halpern Z. Adebayo A. Wing T. Lee A. S. (2019). Intranasal sufentanil versus intravenous morphine for acute pain in the emergency department: A randomized pilot trial. J. Emerg. Med. 56 (3), 301–307. 10.1016/j.jemermed.2018.12.002 30638644

[B40] Slim K. Nini E. Forestier D. Kwiatkowski F. Panis Y. Chipponi J. (2003). Methodological index for non-randomized studies (minors): Development and validation of a new instrument. ANZ J. Surg. 73 (9), 712–716. 10.1046/j.1445-2197.2003.02748.x 12956787

[B41] Smith M. D. Wang Y. Cudnik M. Smith D. A. Pakiela J. Emerman C. L. (2012). The effectiveness and adverse events of morphine versus fentanyl on a physician-staffed helicopter. J. Emerg. Med. 43 (1), 69–75. 10.1016/j.jemermed.2011.05.018 21689900

[B42] Soysal S. Karcioglu O. Demircan A. Topacoglu H. Serinken M. Ozucelik N. (2004). Comparison of meperidine plus midazolam and fentanyl plus midazolam in procedural sedation: A double-blind, randomized controlled trial. Adv. Ther. 21 (5), 312–321. 10.1007/bf02850035 15727400

[B43] Sporer K. A. (2004). Buprenorphine: A primer for emergency physicians. Ann. Emerg. Med. 43 (5), 580–584. 10.1016/s0196064403012058 15111917

[B44] Stang A. (2010). Critical evaluation of the Newcastle-Ottawa scale for the assessment of the quality of nonrandomized studies in meta-analyses. Eur. J. Epidemiol. 25 (9), 603–605. 10.1007/s10654-010-9491-z 20652370

[B45] Stanley T. H. (2014). The fentanyl story. J. Pain 15 (12), 1215–1226. 10.1016/j.jpain.2014.08.010 25441689

[B46] Steenblik J. Goodman M. Davis V. Gee C. Hopkins C. L. Stephen R. (2012). Intranasal sufentanil for the treatment of acute pain in a winter resort clinic. Am. J. Emerg. Med. 30 (9), 1817–1821. 10.1016/j.ajem.2012.02.019 22633713

[B47] Stephen R. Lingenfelter E. Broadwater-Hollifield C. Madsen T. (2012). Intranasal sufentanil provides adequate analgesia for emergency department patients with extremity injuries. J. Opioid Manag. 8 (4), 237–241. 10.5055/jom.2012.0121 22941851

[B48] Trescot A. M. Datta S. Lee M. Hansen H. (2008). Opioid pharmacology. Pain Physician 11 (2), S133–S153. 10.36076/ppj.2008/11/s133 18443637

[B49] Turturro M. A. Paris P. M. Larkin G. L. (1998). Tramadol versus hydrocodone-acetaminophen in acute musculoskeletal pain: A randomized, double-blind clinical trial. Ann. Emerg. Med. 32 (2), 139–143. 10.1016/s0196-0644(98)70127-1 9701294

[B50] Vahedi H. S. M. Hajebi H. Vahidi E. Nejati A. Saeedi M. (2019). Comparison between intravenous morphine versus fentanyl in acute pain relief in drug abusers with acute limb traumatic injury. World J. Emerg. Med. 10 (1), 27–32. 10.5847/wjem.j.1920-8642.2019.01.004 30598715 PMC6264977

[B51] Vergnion M. Degesves S. Garcet L. Magotteaux V. (2001). Tramadol, an alternative to morphine for treating posttraumatic pain in the prehospital situation. Anesth. Analgesia 92 (6), 1543–1546. 10.1097/00000539-200106000-00039 11375843

[B52] Volkow N. D. McLellan A. T. (2016). Opioid abuse in chronic pain--misconceptions and mitigation strategies. N. Engl. J. Med. 374 (13), 1253–1263. 10.1056/NEJMra1507771 27028915

[B53] Wenderoth B. R. Kaneda E. T. Amini A. Amini R. Patanwala A. E. (2013). Morphine versus fentanyl for pain due to traumatic injury in the emergency department. J. Trauma Nurs. 20 (1), 10–15. 10.1097/JTN.0b013e31828660b5 23459426

[B54] Xu N. Chen Q. Huang S. T. Sun K. P. Cao H. (2020). Sufentanil reduces emergence delirium in children undergoing transthoracic device closure of VSD after sevoflurane-based cardiac anesthesia. Braz. J. Cardiovasc. Surg. 35 (5), 660–665. 10.21470/1678-9741-2019-0334 33118730 PMC7598960

[B55] Yin X. Wang X. He C. (2021). Comparative efficacy of therapeutics for traumatic musculoskeletal pain in the emergency setting: A network meta-analysis. Am. J. Emerg. Med. 46, 424–429. 10.1016/j.ajem.2020.10.038 33131973

[B56] Zare M. A. Ghalyaie A. H. Fathi M. Farsi D. Abbasi S. Hafezimoghadam P. (2014). Oral oxycodone plus intravenous acetaminophen versus intravenous morphine sulfate in acute bone fracture pain control: A double-blind placebo-controlled randomized clinical trial. Eur. J. Orthop. Surg. Traumatol. 24 (7), 1305–1309. 10.1007/s00590-013-1392-x 24356922

