# Supplementary figures and images for: Efficacy of opioids for traumatic pain in the emergency department: a systematic review and Bayesian network meta-analysis
Source: Front Pharmacol. 2023 Jul 27;14:1209131. doi: 10.3389/fphar.2023.1209131 (PMC10413574; doi:10.3389/fphar.2023.1209131)

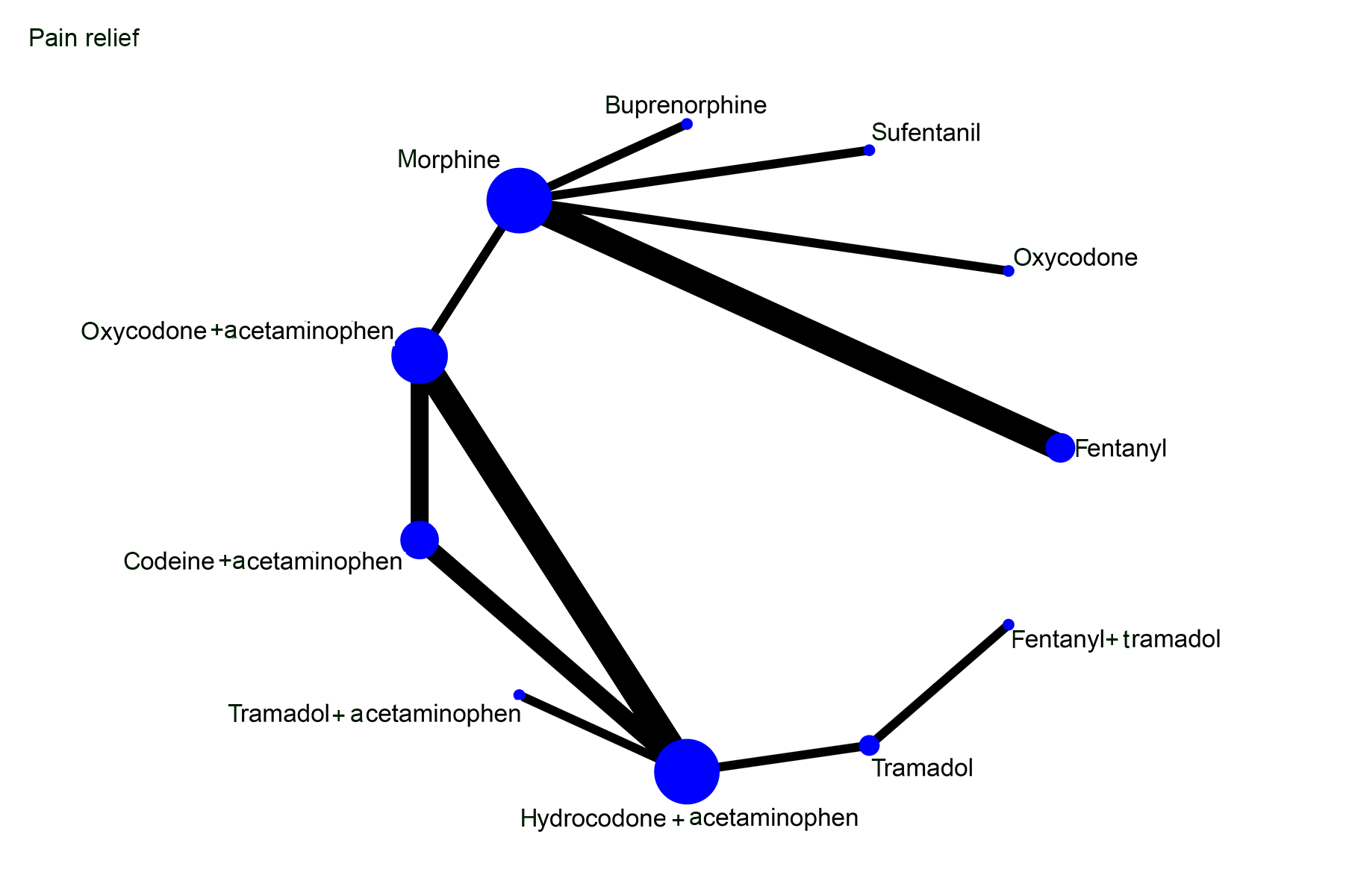

Supplement: Supplementary file 1 [file DataSheet2.zip › Figure S1/Figure S1A.tif]

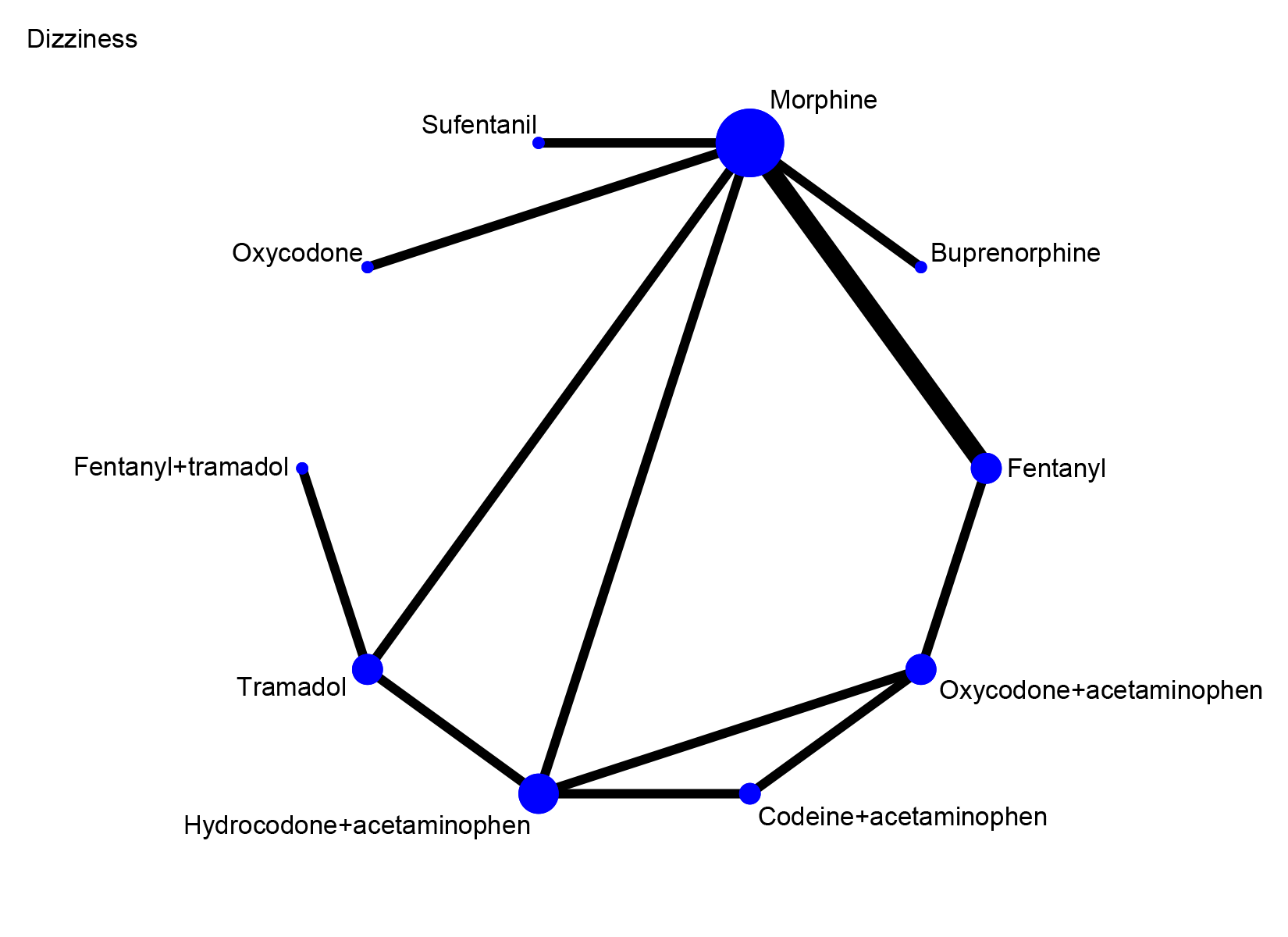

Supplement: Supplementary file 1 [file DataSheet2.zip › Figure S1/Figure S1B.TIF]

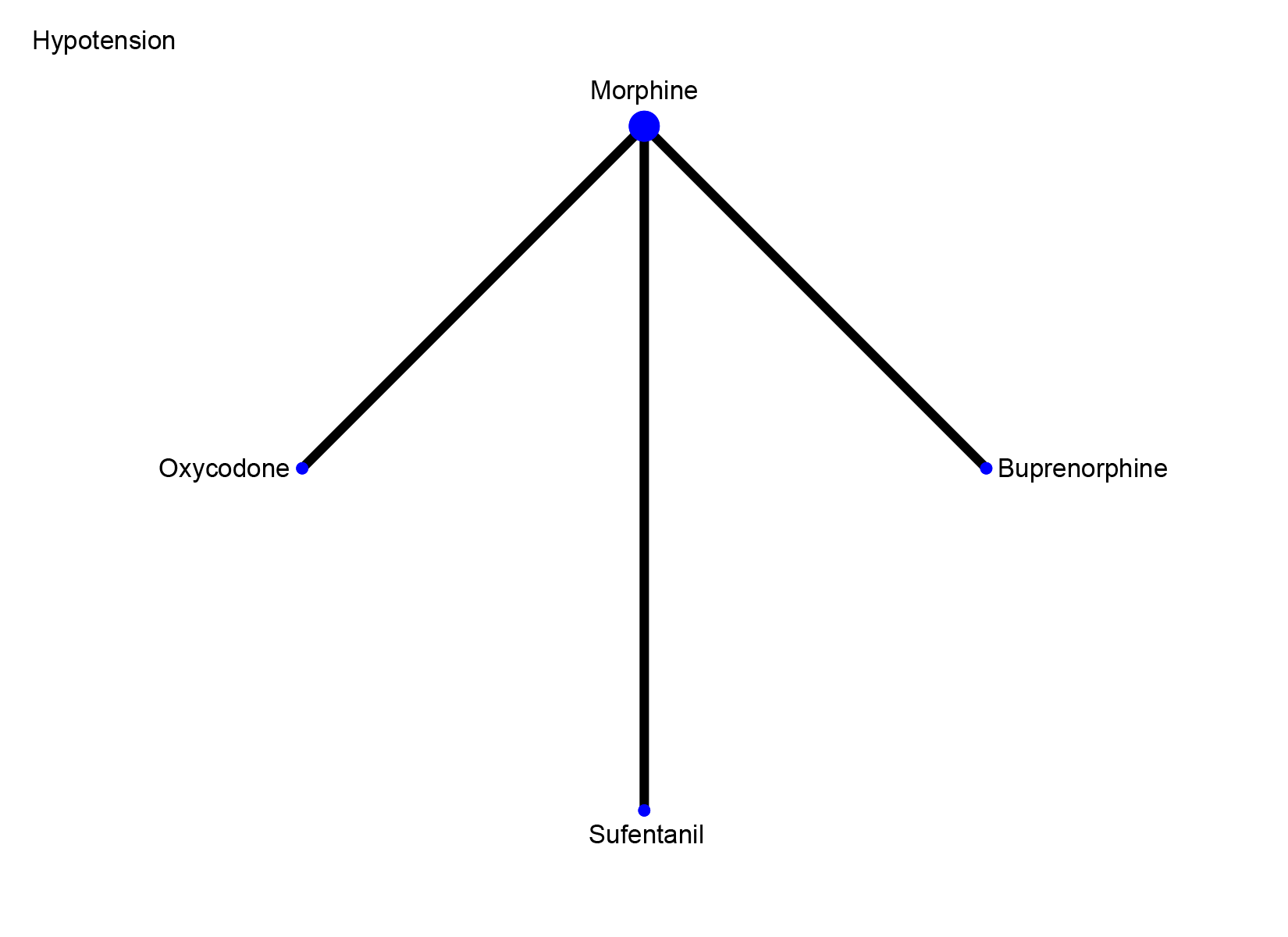

Supplement: Supplementary file 1 [file DataSheet2.zip › Figure S1/Figure S1C.TIF]

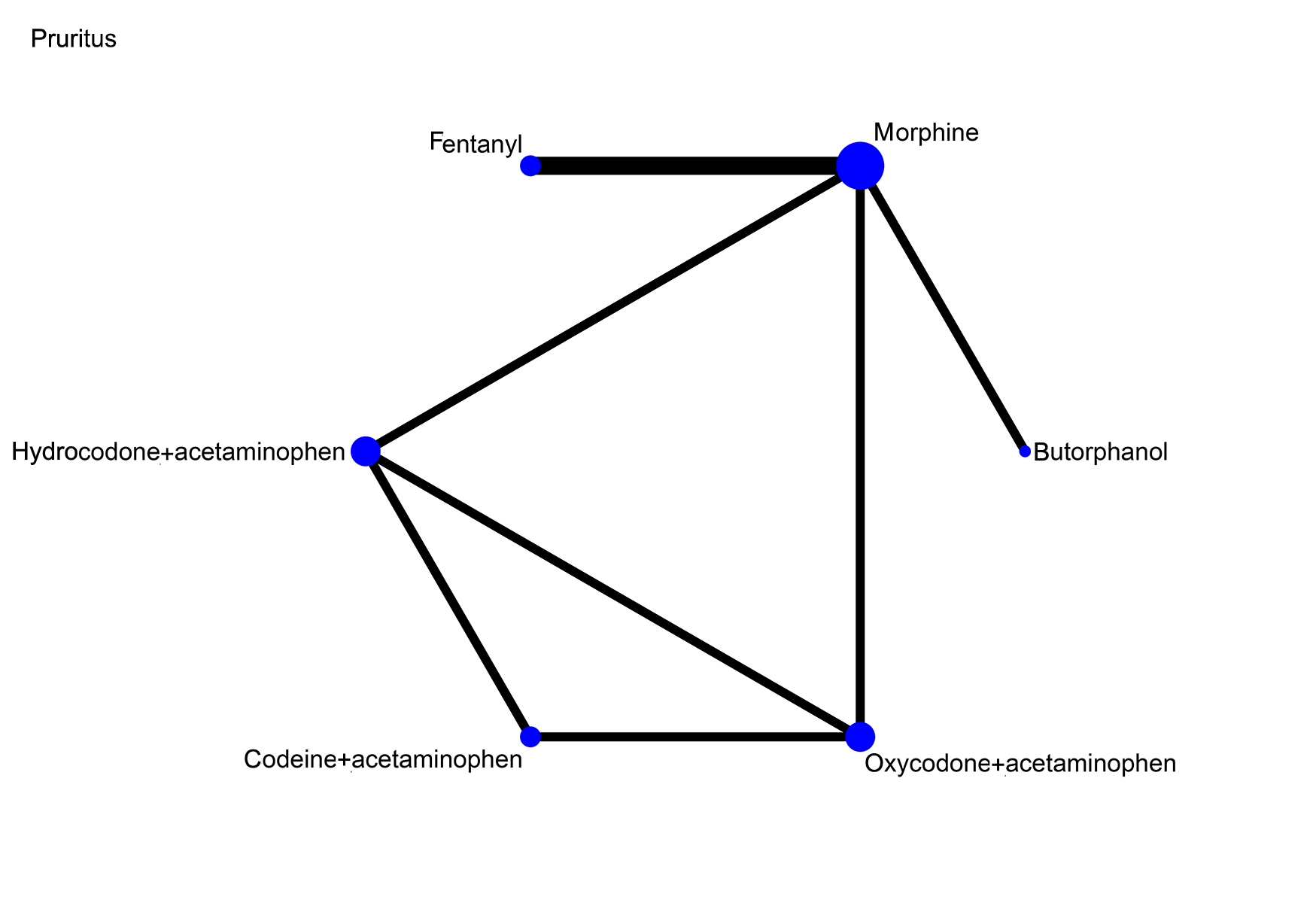

Supplement: Supplementary file 1 [file DataSheet2.zip › Figure S1/Figure S1D.TIF]

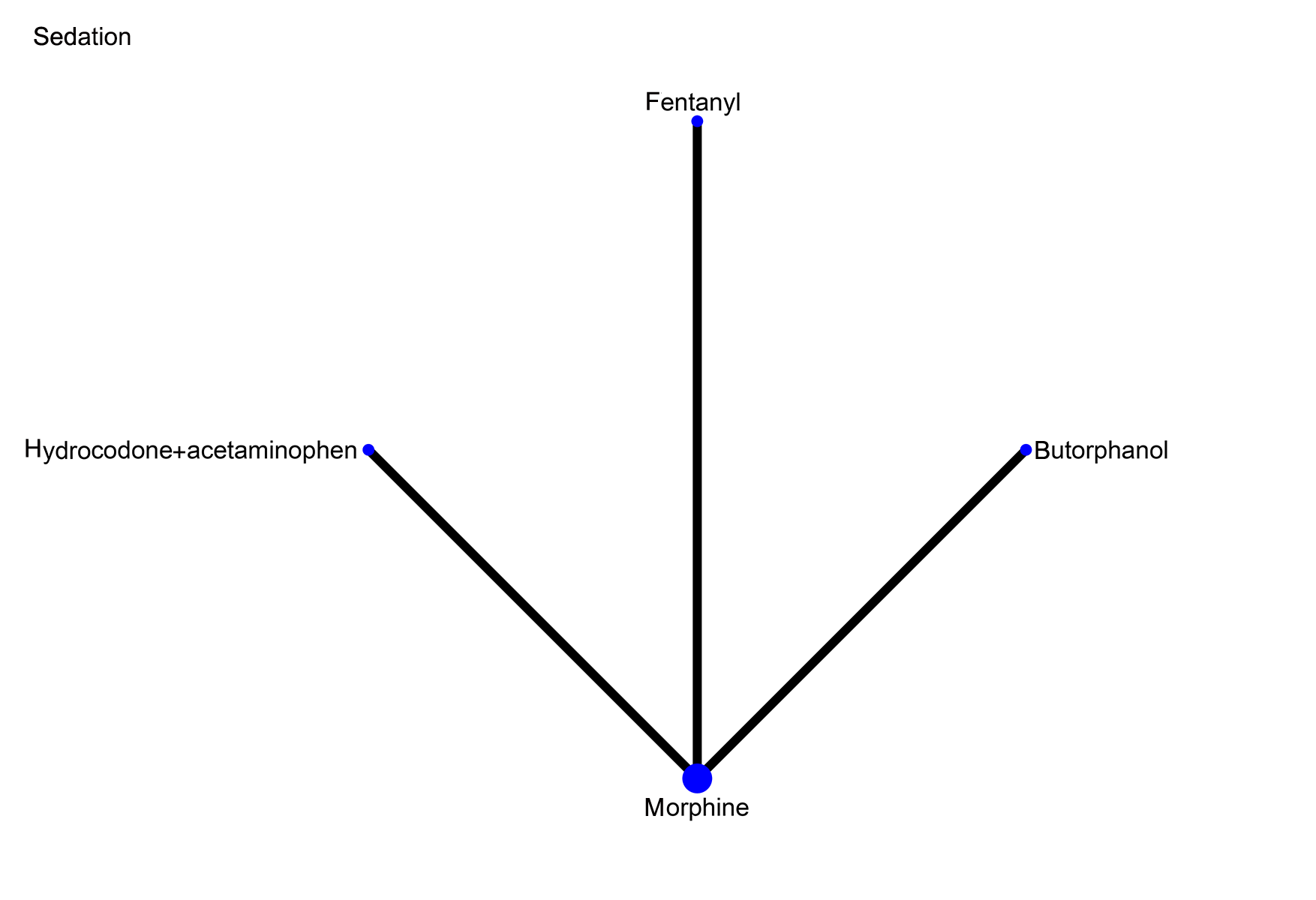

Supplement: Supplementary file 1 [file DataSheet2.zip › Figure S1/Figure S1E.TIF]

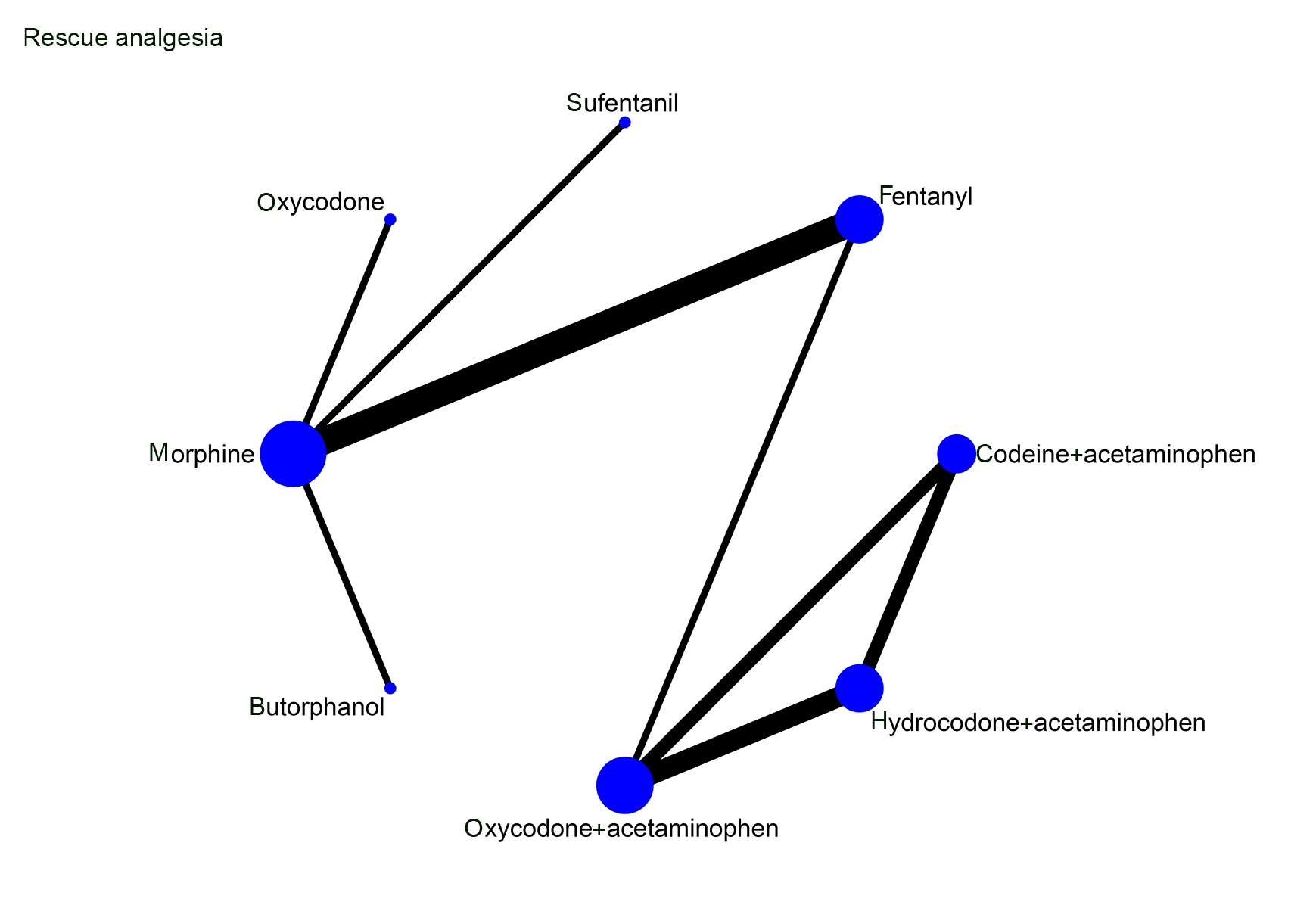

Supplement: Supplementary file 1 [file DataSheet2.zip › Figure S1/Figure S1F.TIF]

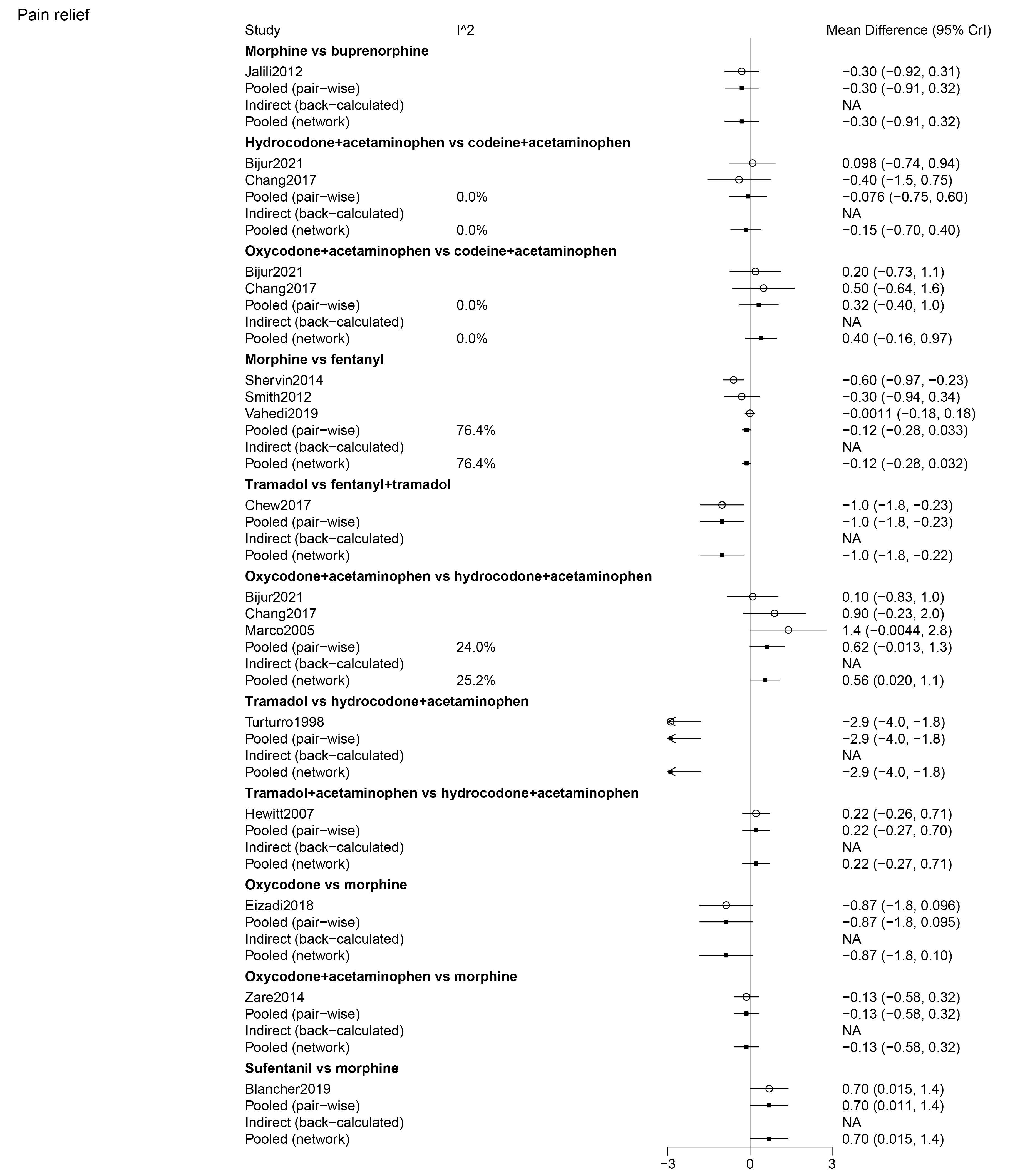

Supplement: Supplementary file 1 [file DataSheet2.zip › Figure S2/Figure S2A.TIF]

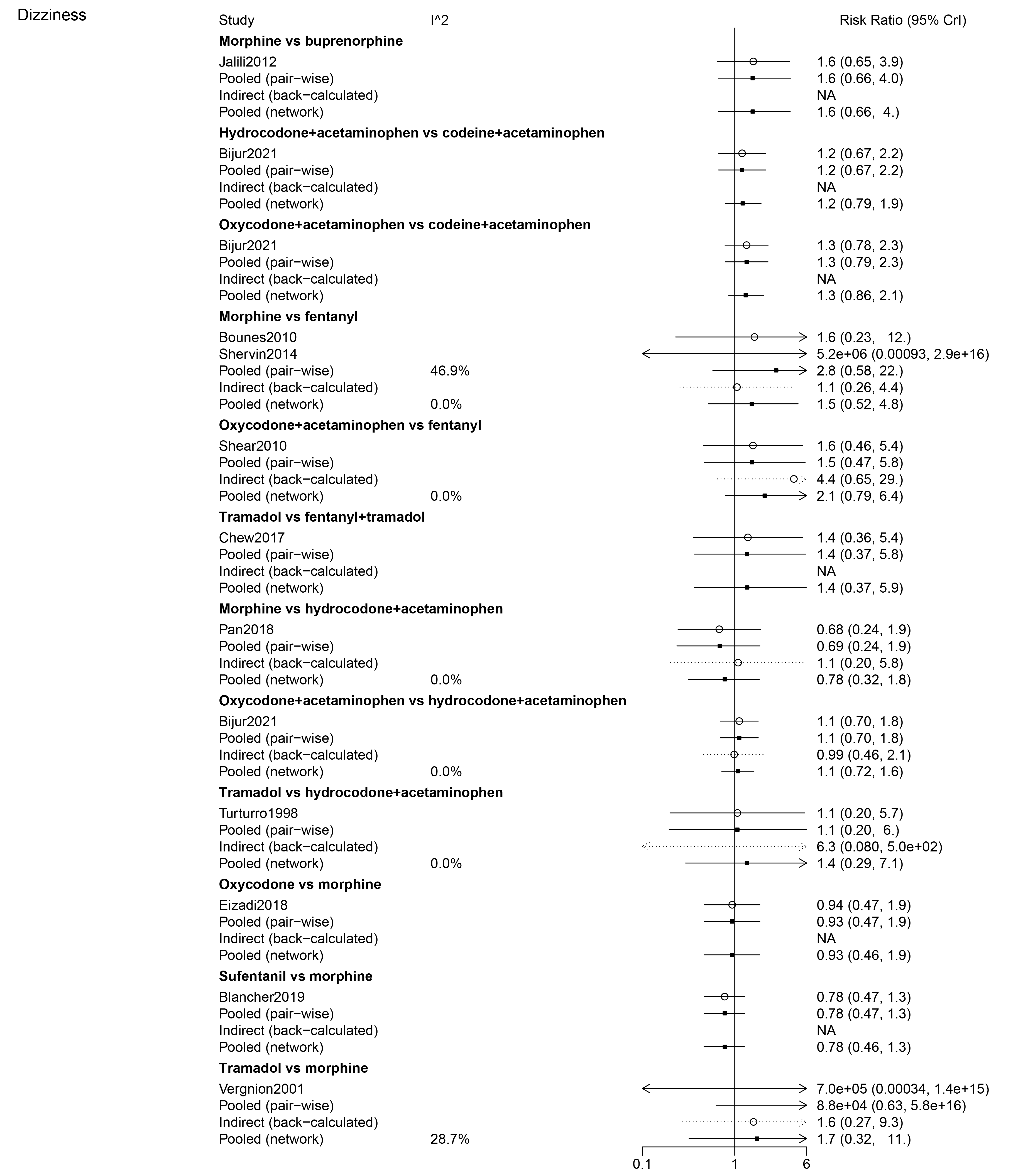

Supplement: Supplementary file 1 [file DataSheet2.zip › Figure S2/Figure S2B.TIF]

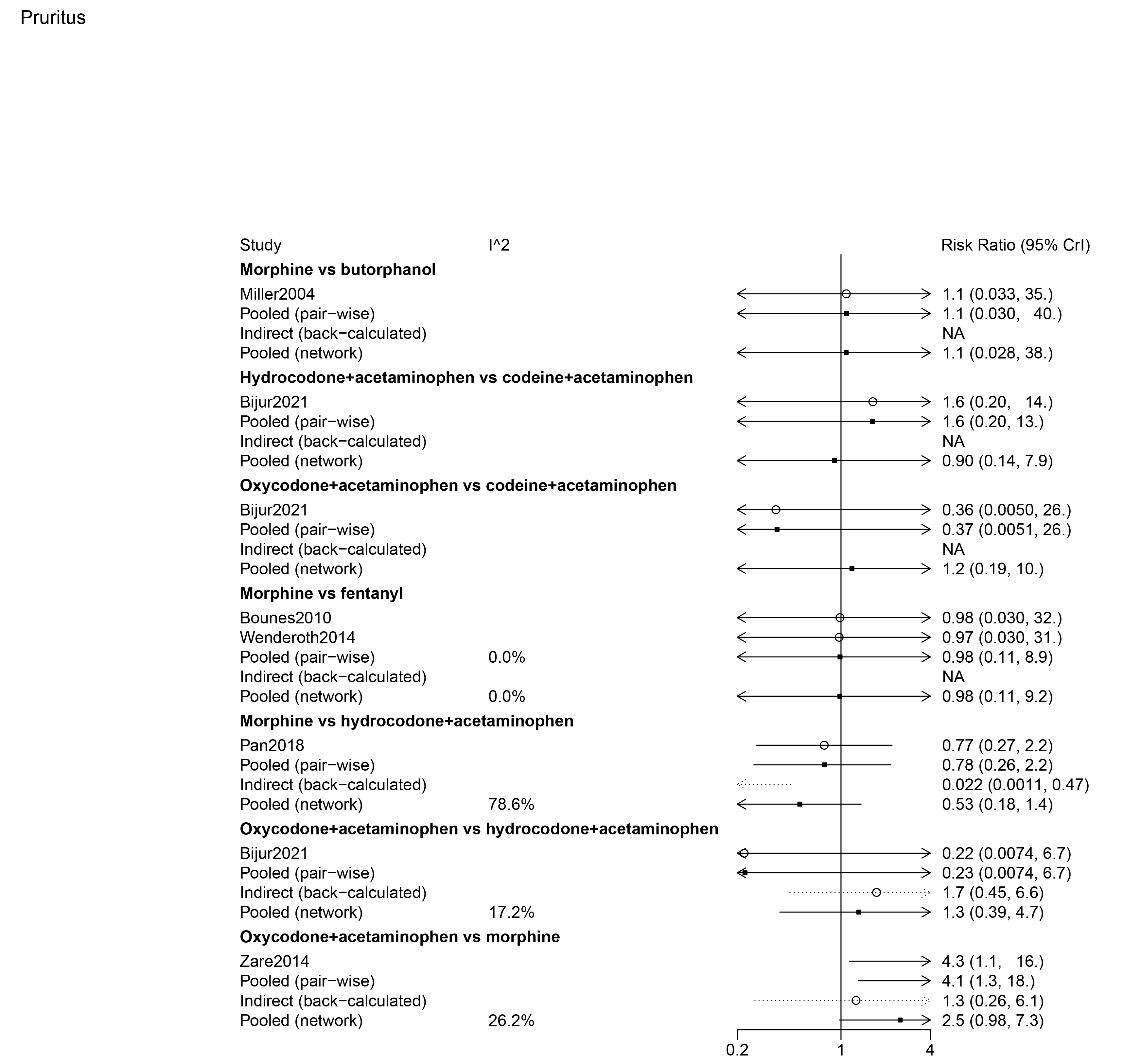

Supplement: Supplementary file 1 [file DataSheet2.zip › Figure S2/Figure S2C.TIF]

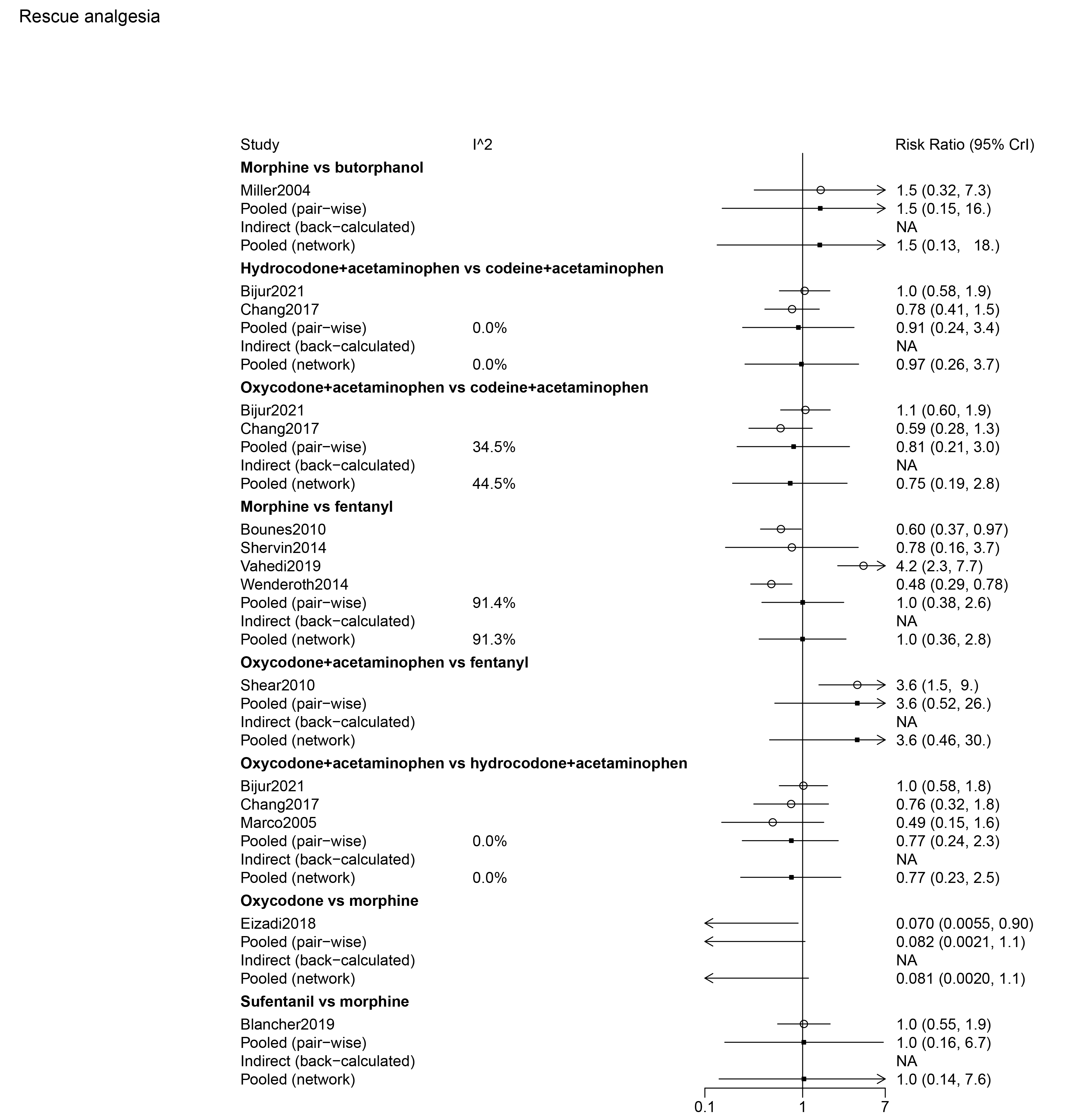

Supplement: Supplementary file 1 [file DataSheet2.zip › Figure S2/Figure S2D.TIF]

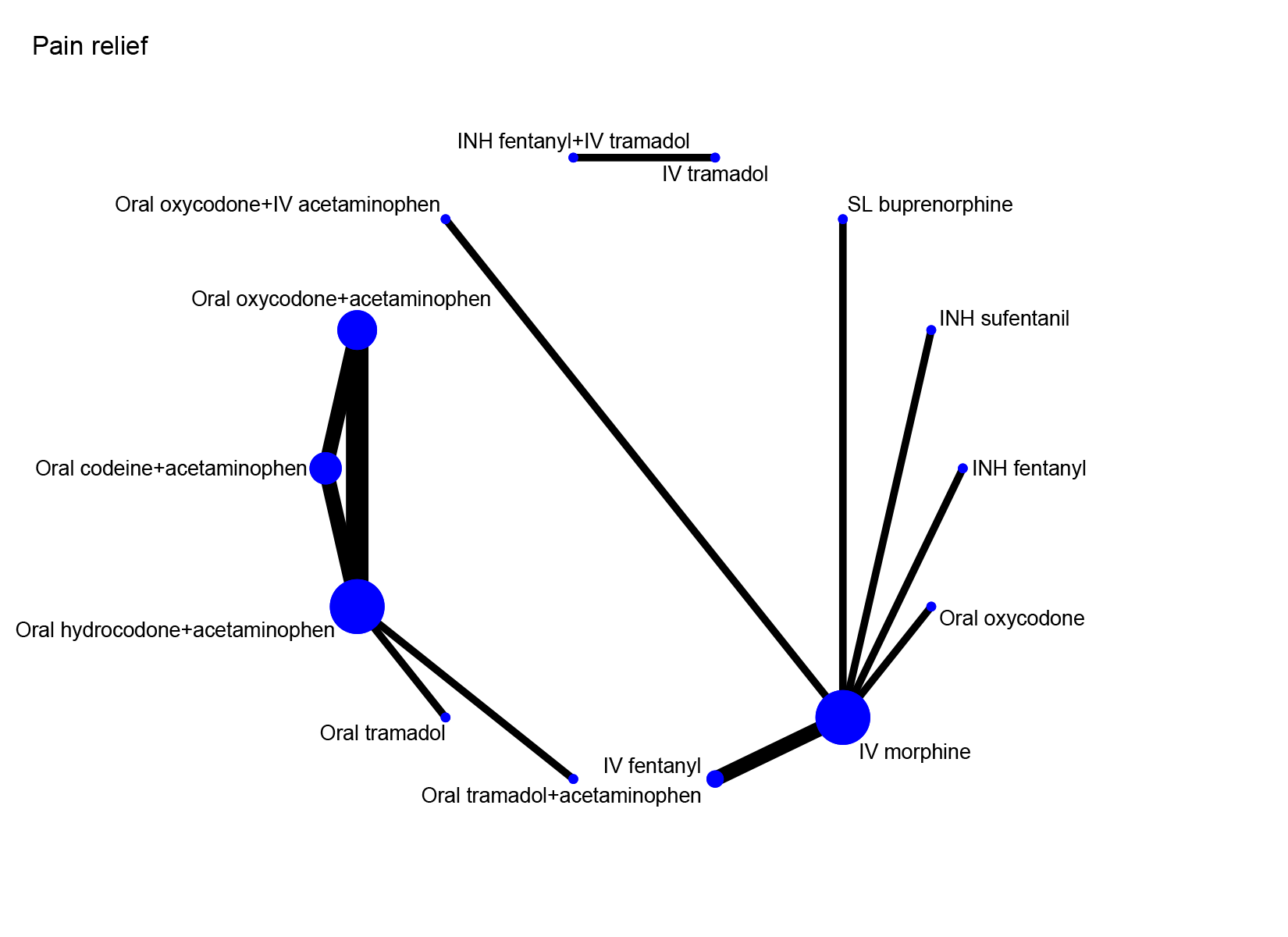

Supplement: Supplementary file 1 [file DataSheet2.zip › Figure S3.TIF]
