# Supplementary material for: Efficacy of opioids for traumatic pain in the emergency department: a systematic review and Bayesian network meta-analysis
Source: Front Pharmacol. 2023 Jul 27;14:1209131. doi: 10.3389/fphar.2023.1209131 (PMC10413574; doi:10.3389/fphar.2023.1209131)
Supplement: Supplementary file 2 [file DataSheet1.docx]

Supplementary Table 1 Basic characteristics of the included studies.

| Author | Year | Country | Study design | Study population (indication) | Drug administration and type | Dose | Number of participants | Sex (M/F) | Age (years) | Pain score | Type of injury | FU | Outcome |
| --- | --- | --- | --- | --- | --- | --- | --- | --- | --- | --- | --- | --- | --- |
| Bijur | 2021 | USA | Double-blind RCT | Aged 21 through 64 years, had a complaint of acute musculoskeletal pain in one or more extremities | Oral oxycodone/acetaminophen | 5 mg/325 mg | 120 | 67/53 | 36±12 | 0-6 5, 7-10 115 | Sprain or strain 75, extremity fracture 21, muscle pain 12, contusion 1, other 11 | 2 h | Pain relief, dizziness, pruritus, rescue analgesia |
|  |  |  |  |  | Oral hydrocodone/acetaminophen | 5 mg/300 mg | 119 | 69/50 | 39±12 | 0-6 4, 7-10 115 | Sprain or strain 78, extremity fracture 11, muscle pain 13, contusion 10, other 7 |  |  |
|  |  |  |  |  | Oral codeine/acetaminophen | 30 mg/300 mg | 120 | 61/59 | 38±12 | 0-6 1, 7-10 119 | Sprain or strain 68, extremity fracture 17, muscle pain 17, contusion 11, other 7 |  |  |
| Blancher | 2019 | France | Double-blind RCT | Adult patients (18 to 75 years old) presenting with traumatic pain self-evaluated as ≥6/10 on a NRS | IV morphine | Initial 0.1 mg/kg, additional 0.05 mg/kg | 69 | 40/29 | 41 (28-54)* | 8 (7-8)* | - | 0.5 h | Pain relief, hypotension, dizziness, rescue analgesia |
|  |  |  |  |  | IN sufentanil | Initial 0.3 μg/kg, additional 0.15 μg/kg | 67 | 31/36 | 38 (30-55)* | 8 (7-9)* |  |  |  |
| Vahedi | 2019 | Iran | Double-blind RCT | >18 years old with pain score more than 5, suffer from acute traumatic limb injuries and addicted to opioids | IV morphine | 0.1 mg/kg | 152 | 141/11 | 31.8±10.4 | 6.8±0.8 | Acute traumatic limb injuries | 1 h | Pain relief, rescue analgesia |
|  |  |  |  |  | IV fentanyl | 1 mcg/kg | 155 | 138/17 | 31.0±10.7 | 6.8±0.8 |  |  |  |
| Eizadi | 2018 | Iran | Double-blind, placebo-controlled RCT | Aged 14 or above with moderate to severe pain(NRS>3) following isolated limb trauma | IV morphine sulfate | 5 mg | 28 | 23/5 | 32.86±15.39 | 8.32±1.36 | Isolated limb trauma | 1 h | Pain relief, hypotension, dizziness, rescue analgesia |
|  |  |  |  |  | Oral oxycodone | 5 mg | 30 | 20/10 | 29.27±9.35 | 7.53±1.55 |  |  |  |
| Pan | 2018 | China | RCT | 18-65 years old healthy adults, suspected lower extremity displaced fracture within 2h after trauma, moderate or severe pain (VAS≥70) with a pulse oxygen saturation of ≥95% and a respiratory rate of ≥12 breaths/min | IV morphine | Titrated every 5 min by 3-mg increments | 81 | 62/19 | 43.8±8.2 | 8.91±0.56 | Fracture | 2 h | Dizziness, pruritus, sedation |
|  |  |  |  |  | Oral hydrocodone/acetaminophen | 5 mg/500 mg | 85 | 61/24 | 45.2±8.7 | 8.80±0.68 |  |  |  |
| Chang | 2017 | USA | Double-blind RCT | Adults aged 21 years through 64 years, presented to the ED for management of acute extremity pain | Oral oxycodone/acetaminophen | 5 mg/325 mg | 104 | 54/50 | 37±12 | 8.7±1.3 | Sprain or strain 66, extremity fracture 23, muscle pain 9, contusion 3, other 3 | 2 h | Pain relief, rescue analgesia |
|  |  |  |  |  | Oral hydrocodone/acetaminophen | 5 mg/300 mg | 103 | 52/51 | 37±13 |  | Sprain or strain 59, extremity fracture 21, muscle pain 12, contusion 7, other 4 |  |  |
|  |  |  |  |  | Oral codeine/acetaminophen | 30 mg/300 mg | 103 | 59/44 | 37±12 |  | Sprain or strain 67, extremity fracture 24, muscle pain 7 contusion 2, other 3 |  |  |
| Chew | 2017 | Malaysia | Open-label RCT | Adult patients (i.e. Aged ≥ 18 years) with moderate to severe pain due to musculoskeletal injuries | IN fentanyl/IV tramadol | 1.5 mcg/kg/2 mg/kg | 10 | 8/2 | 18-65 | ≥4.5 | Soft tissue injury + bone fractures 4, soft tissue injury 6 | 1-2 h | Pain relief, dizziness |
|  |  |  |  |  | IV tramadol | 2 mg/kg | 10 | 8/2 |  |  | Soft tissue injury + bone fractures 3, soft tissue injury 7 |  |  |
| Shervin | 2014 | Iran | Double-blind, placebo-controlled RCT | Aged 15 to 50 years presenting to the ED due to limb trauma, with acute pain with NRS score above 5 | Nebulized fentanyl | 4 μg/kg | 47 | 39/8 | 26.80±7.45 | 8.7±1.0 | Wound and soft tissue injuries 8, fractures 23, sprains and strains 16 | 1 h | Pain relief, dizziness, rescue analgesia |
|  |  |  |  |  | IV morphine | 0.1 mg/kg | 43 | 36/7 | 26.86±7.73 | 8.4±0.8 | Wound and soft tissue injuries 15, fractures 18, sprains and strains 10 |  |  |
| Zare | 2014 | Iran | Double-blind, placebo-controlled RCT | 15–60 years old, with acute bone fracture(s) and a pain VAS higher than 5 of 10 | IV morphine sulfate | 5 mg | 74 | 62/12 | 31.4±4.1 | 7.32±1.4 | Bone fracture | 1 h | Pain relief, pruritus |
|  |  |  |  |  | Oral oxycodone/IV acetaminophen | 10 mg/1 g | 79 | 64/15 | 31.5±3.9 | 7.38±1.4 |  |  |  |
| Wenderoth | 2013 | USA | Retrospective cohort | Adult trauma patients brought to the ED, with an initial pain score of 4 or more | IV morphine | 4 mg | 84 | 56/28 | 37 (24-51)* | 8 (7-9.5)* | Blunt 72, penetrating 12 | 2 h | Pruritus, rescue analgesia |
|  |  |  |  |  | IV fentanyl | 50 μg | 84 | 57/27 | 38 (24-53)* | 10 (8-10)* | Blunt 70, penetrating 14 |  |  |
| Jalili | 2012 | Iran | Double-blind, placebo-controlled RCT | 16 years or older, with acute extremity fracture(s) and a pain NRS score higher than 3 of 10 | IV morphine sulfate | 5 mg | 55 | 45/10 | 35±13 | 7.7±1.7 | Bone fracture | 1 h | Pain relief, hypotension, dizziness |
|  |  |  |  |  | Sublingual buprenorphine | 0.4 mg | 55 | 44/11 | 35±13 | 8.0±1.7 |  |  |  |
| Smith | 2012 | USA | Non-randomized clinical trial | Ages of 18 and 65 years transported by helicopter for evaluation of traumatic injuries | IV morphine sulfate | 4 mg | 104 | 78/26 | 38.5±13 | 8.0±2.0 | Blunt 94, penetrating 10 | - | Pain relief |
|  |  |  |  |  | IV fentanyl | 50 μg | 100 | 76/24 |  | 8.0±1.8 | Blunt 85, penetrating 15 |  |  |
| Bounes | 2010 | France | Double-blind RCT | Aged 18 years or older, with acute severe pain (NRS score of 6/10 or higher) caused by trauma | IV morphine | 0.15 mg/kg followed by 0.075 mg/kg every 3 minutes until pain relief | 54 | 32/22 | 44 (30–61)* | 8 (7–10)* | Fracture 23, dislocation 13, soft tissue injury 9, back trauma 3, other 6 | 6 h | Dizziness, pruritus, sedation, rescue analgesia |
|  |  |  |  |  | IV fentanyl | 0.15 μg/kg followed by 0.075 ug/kg every 3 minutes until pain relief | 54 | 40/14 | 47 (29–65)* | 8 (7–9)* | Fracture 28, dislocation 12, soft tissue injury 4, back trauma 7, other 3 |  |  |
| Shear | 2010 | USA | Double-blind trial | 18 to 60 years, with a chief complaint of extremity injury | Transbuccal fentanyl | 100 μg | 30 | 20/10 | 40 (29-49)* | 9 (8-10)* | Fracture or dislocation 11 | 1 h | Dizziness, rescue analgesia |
|  |  |  |  |  | Oral oxycodone/acetaminophen | 5 mg/325 mg | 30 | 18/12 | 34 (27-45)* | 8 (8-10)* | Fracture or dislocation 15 |  |  |
| Hewitt | 2007 | USA | Double-blind, placebo-controlled RCT | Adults (18 to 75 years) with ankle sprain within the previous 48 hours, have a pain score of greater than or equal to 50 mm on a VAS from 0 mm (no pain) to 100 mm (extreme pain) | Oral hydrocodone/acetaminophen | 7.5 mg/650 mg | 201 | 114/87 | 33.6±12.8 | 7.4±1.2 | Ankle sprain | 4 h | Pain relief |
|  |  |  |  |  | Oral tramadol/acetaminophen | 75 mg/650 mg | 190 | 90/100 | 30.7±11.8 | 7.6±1.2 |  |  |  |
| Marco | 2005 | USA | Double-blind RCT | Adult and adolescent patients with acute fracture (less than three days) and severe pain, with pain scores ≥5 on a 0–10 scale | Oral oxycodone/acetaminophen | 5 mg/325 mg | 35 | 22/13 | 35.6±10.7 | 7.7±1.7 | Fracture | 1 h | Pain relief, rescue analgesia |
|  |  |  |  |  | Oral hydrocodone/acetaminophen | 5 mg/325 mg | 32 | 20/12 | 36.0±12.4 | 7.1±2.0 |  |  |  |
| Miller | 2004 | USA | Double-blind RCT | 18 to 65 years of age, moderate to severe pain associated with acute injury and require IV medication for pain control | IV morphine sulfate | 2.5-5 mg | 46 | 24/22 | 38 | 7.6 (6.8-8.5)* | Acute uncomplicated fractures, dislocations, severe sprains, or other isolated injuries | 2 h | Pruritus, sedation, rescue analgesia |
|  |  |  |  |  | IV butorphanol | 0.5-1 mg | 48 | 25/23 | 41 | 7.6 (6.0-8.6)* |  |  |  |
| Soysal | 2004 | Turkey | Rct | Adult patients with extremity fracture or dislocation | IV meperidine/IV midazolam | 0.5 mg/kg/0.02 mg/kg | 34 | 14/20 | 42.94±16.88 | 7.1±2.1 | Extremity fracture or dislocation | - | Rescue analgesia |
|  |  |  |  |  | IV fentanyl/IV midazolam | 1 mcg/kg/0.02 mg/kg | 36 | 16/20 | 45.58±16.51 | 6.0±2.6 |  |  |  |
| Vergnion | 2001 | Belgium | Double-blind RCT | Posttraumatic musculoskeletal pain requiring rapid analgesia with opioids | IV morphine | 5-20 mg | 48 | - | - | - | Musculoskeletal pain | ≥0.67 h | Dizziness |
|  |  |  |  |  | IV tramadol | 100-200 mg | 53 |  |  |  |  |  |  |
| Turturro | 1998 | USA | Double-blind RCT | Aged 18 to 70 years, with acute musculoskeletal pain caused by trauma | Oral tramadol | 100 mg | 33 | - | - | 6.8±2.2 | Fracture 9, sprain/strain 15, contusion 9 | 3 h | Pain relief, dizziness |
|  |  |  |  |  | Oral hydrocodone/acetaminophen | 5 mg/500 mg | 35 |  |  | 6.9±1.8 | Fracture 11, sprain/strain 11, contusion 12, tendon rupture 1 |  |  |

* Median (interquartile range).

ED, emergency department; M/F, male/female; FU, follow-up time; IN, intranasal; IV, intravenous; NRS, Numerical Rating Scale; RCT, randomized clinical trial; VAS, Visual Analogue Scale.

Supplementary Table 2 GRADE approach for quality of evidence evaluation in this network meta-analysis.

| **Certainty assessment** | | | | | | | **Certainty** | **Importance** |
| --- | --- | --- | --- | --- | --- | --- | --- | --- |
| **Number of studies** | **Study design** | **Risk of bias** | **Inconsistency** | **Indirectness** | **Imprecision** | **Other considerations** |  |  |
| **Pain relief** | | | | | | | | |
| 13 | Randomised trials | Not serious | Not serious | Serious^a^ | Not serious | None | ⨁⨁⨁◯ Moderate | CRITICAL |
| **Dizziness** | | | | | | | | |
| 11 | Randomised trials | Serious^c^ | Not serious | Not serious | Not serious | None | ⨁⨁⨁◯ Moderate | IMPORTANT |
| **Hypotension** | | | | | | | | |
| 3 | Randomised trials | Serious^e^ | Not serious | Not serious | Serious^d^ | None | ⨁⨁◯◯ Low | NOT IMPORTANT |
| **Pruritus** | | | | | | | | |
| 6 | Randomised trials | Serious^c^ | Not serious | Not serious | Serious^d^ | None | ⨁⨁◯◯ Low | IMPORTANT |
| **Sedation** | | | | | | | | |
| 3 | Randomised trials | Serious^c^ | Not serious | Serious^a^ | Not serious | None | ⨁⨁◯◯ Low | NOT IMPORTANT |
| **Rescue analgesia** | | | | | | | | |
| 11 | Randomised trials | Not serious | Not serious | Serious^a^ | Serious^d^ | None | ⨁⨁◯◯ Low | IMPORTANT |

^a^ Indirect outcomes of some groups are not available; ^b^ I²>50%; ^c^ Randomization and blinding of some studies are not clear; ^d^ Large 95% confidence interval; ^e^ incomplete outcome data.

GRADE, Grading of Recommendations Assessment, Development, and Evaluation.
